# Rhizosphere Effect Enhances Belowground Competition of Coastal Invasive *Spartina alterniflora* With Mangroves

**DOI:** 10.1002/ece3.72565

**Published:** 2025-11-30

**Authors:** Dandan Long, Wentao Zhao, Xishuai Li, Qing Sun, Jiqiu Li, Xiaofeng Lin

**Affiliations:** ^1^ Key Laboratory of Ministry of Education for Coastal and Wetland Ecosystems, Fujian Province Key Laboratory for Coastal Ecology and Environmental Studies, College of the Environment and Ecology Xiamen University Xiamen China; ^2^ State Key Laboratory of Marine Environmental Science, National Observation and Research Station for the Taiwan Strait Marine Ecosystem Xiamen University Xiamen China

**Keywords:** interspecific interaction, metagenomic, rhizosphere microbiome, root exudates, soil nutrient cycling

## Abstract

*Spartina alterniflora* has severely invaded mangroves in China. In order to explore the possible belowground interspecific interaction along with its invasion, the rhizosphere effect enhancing the competition of *S. alterniflora* neighboring mangroves was hypothesized. Here, both rhizosphere soil of *S. alterniflora* and bulk soil were collected from the center of *S. alterniflora* marsh and border sites where *S. alterniflora* was adjacent to *Kandelia obovata* and *Aegiceras corniculatum*, respectively, in both vigorous growth and senescent periods. Soil nutrient properties, rhizospheric low‐molecular‐weight organic acids (LMWOAs), soil microbiomes, and microbial functional genes were analyzed. Soil total carbon and total nitrogen contents of *S. alterniflora* neighboring mangroves were increased, and its LMWOAs were altered when adjacent to mangroves in both vigorous growth and senescent periods. These changes were significantly correlated with variation in the composition of *S. alterniflora* rhizosphere microbiome. Microbial interkingdom co‐occurrence networks were simplified when *S. alterniflora* neighbored mangroves, while network modularity significantly increased. Metagenomics indicated that genes involved in methanogenesis (*ackA*, *mvhD*, etc.) and nitrogen fixation (*nifH*, *nifK*, etc.) were significantly enriched in those *S. alterniflora* neighboring *K. obovata*, and genes related to phosphate transporter (*pstA*, *pstB*, etc.) were significantly enriched in those *S. alterniflora* neighboring *A. corniculatum*. These results demonstrated that the rhizosphere effect intensified the belowground interspecific competition of *S. alterniflora* adjacent to mangroves by altering root exudates, changing the soil microbial composition, and modulating strategies for core nutrient metabolism.

## Graphical abstract

This work demonstrated that the rhizosphere effect intensified the belowground interspecific competition of invasive *Spartina alterniflora* adjacent to mangroves by altering root exudates, changing soil microbial composition, and modulating some core nutrient metabolism strategies.

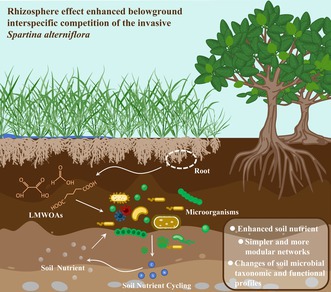

## 1 Introduction

As an important driver of community assembly and evolutionary change, plant interspecific interactions have drawn attention in diverse ecosystems (Delic and Fiser 2019; Gaudio et al. 2021). The aboveground interactions of vegetation are mainly for light (Bloor et al. 2007; Ding et al. 2016), while in the belowground, they may compete fiercely for nutrients and water (Coomes and Grubb 2000; Passarge et al. 2006). The rhizosphere is the narrow zone of soil surrounding the roots of plants (Prashar et al. 2014). Under the influence of living plant roots, the rhizosphere soil showed huge differences from the bulk soil, including soil properties, soil microbes, and soil processes, which was termed the rhizosphere effect (Phillips and Fahey 2006; Ding et al. 2019). The plant–soil–microbe interactions occurring in the rhizosphere play important roles in plants' belowground interactions (Bais et al. 2004, 2006; Guerrieri and Rasmann 2024).

For example, allelopathy mediated by root exudates helps some plants gain advantages over their competitors (Callaway and Aschehoug 2000; Zhang et al. 2016; Xu et al. 2021). Meanwhile, some exudates, acting as signaling chemicals, can drive plant neighbor recognition and response (Kong et al. 2018; Wang et al. 2021). Among them, low‐molecular‐weight organic acids (LMWOAs) are involved in various biochemical processes in the rhizosphere, including plant defense, nutrient acquisition, plant‐microbe interactions, and so on (Jones 1998; Bais et al. 2006; Zhao et al. 2024). They are essential carbon sources for bacteria (Campbell et al. 1997; Gunina et al. 2014), and the shifts of LMWOAs and other root exudates could affect the composition and function of soil microbiomes (Chaparro et al. 2013; Lebeis et al. 2015).

Soil microbiomes are important for the growth and development of plants (Trivedi et al. 2020). They are also closely associated with plant interspecific competition via litter decomposition and nutrient cycling (Elgersma et al. 2012; Knelman et al. 2018). On the one hand, the outcome of plant competition can be modulated by soil microbial communities (Hortal et al. 2017; Siefert et al. 2018). Soil microbes can affect the niche difference between competing plants (Ke and Wan 2020), and microbe‐responsive plant species may have higher competitive ability than non‐responsive plants with increasing rhizobia and mycorrhizal fungi (Abbott et al. 2015). On the other hand, soil microbes can be modified through plant belowground interactions (Reinhart and Callaway 2006; Zhou et al. 2023). To become more competitive, invasive plants can alter the composition of soil microbial communities and enhance positive soil feedback (Zhou et al. 2017; Keet et al. 2021). However, the influence of plant interspecific interactions on soil microbial interkingdom interactions remains unclear, although it may influence plant survival (Duran et al. 2018).

Meanwhile, microbial functional genes have usually been used to predict nutrient cycling processes (Li et al. 2019; Pressler et al. 2020). Field experiments have shown that plant intercropping can influence soil microbial communities, which can further alter the abundance of functional genes related to carbon, nitrogen, phosphorus, and sulfur metabolism, and thus change soil nutrient cycling (Shu et al. 2024; You et al. 2024). Furthermore, plant invasion is another essential driver of the variations in microbial functional genes and soil nutrient dynamics (Ehrenfeld et al. 2001; Zhao et al. 2023), while shifts in soil nutrient properties can in turn alter microbiomes and facilitate plant invasion (Lazzaro et al. 2014; Gibbons et al. 2017).

As an invasive plant, *Spartina alterniflora* has widely spread in Chinese coastal zones and invaded native mangroves since it was introduced to China in 1979 (Li et al. 2014; Gao et al. 2022). Like most successful invasive species, *S. alterniflora* possesses multiple invasion strategies, such as high salt tolerance (Tang et al. 2014), genotypic diversity (Wang et al. 2012), and phenotypic plasticity (Liu et al. 2016). The ecological impacts caused by its invasion have been well studied, including the variations of soil physicochemical properties (Yang et al. 2016), microbiomes (Gao et al. 2019), and biogeochemical cycles (Bu et al. 2018; Chen et al. 2023) by comparison with unvegetated bare mudflats or native vegetation. However, little is known about the belowground competition of *S. alterniflora* with other plants, especially the roles played by its root exudates and rhizosphere microorganisms, except that a few studies have indicated that its root exudates have an allelopathic effect on native plants (Zheng et al. 2011; Liang et al. 2012).

Here, in order to explore the belowground competition that *S. alterniflora* may have when invading mangroves, a field investigation was conducted. We hypothesized that *S. alterniflora* neighboring mangroves would exhibit the corresponding belowground competitive responses through its rhizosphere effects. To test the hypothesis, we chose *Kandelia obovata* and *Aegiceras corniculatum* as the neighboring mangroves and investigated a series of environmental factors including soil nutrient properties, low‐molecular‐weight organic acids, and both bacterial and fungal communities not only in the vigorous growth period but also in the senescent period. Furthermore, metagenomic sequencing was performed to investigate the functional profiles of microorganisms. We hope this study can provide further insight into the invasion strategies of *S. alterniflora* .

## 2 Materials and Methods

### 2.1 Study Site Description and Sample Collection

This study was carried out in Zhangjiang Estuary Mangrove National Nature Reserve, Fujian Province, China. With a subtropical marine climate, the annual mean air temperature there is 21.5°C and the annual mean rainfall is 1714.5 mm (Zhang et al. 2006). This reserve is affected by irregular semidiurnal tides, and the mean tidal range is 2.3 m. The dominant vegetation includes *Kandelia obovata*, *Aegiceras corniculatum*, *Avicennia marina* , and *Spartina alterniflora* (Chen et al. 2020). The invasive *S. alterniflora* has rapidly spread in Zhangjiang Estuary through seed dispersal and intentional planting since the late 1990s (Liu et al. 2017; Zhu et al. 2019).

**FIGURE 1:**
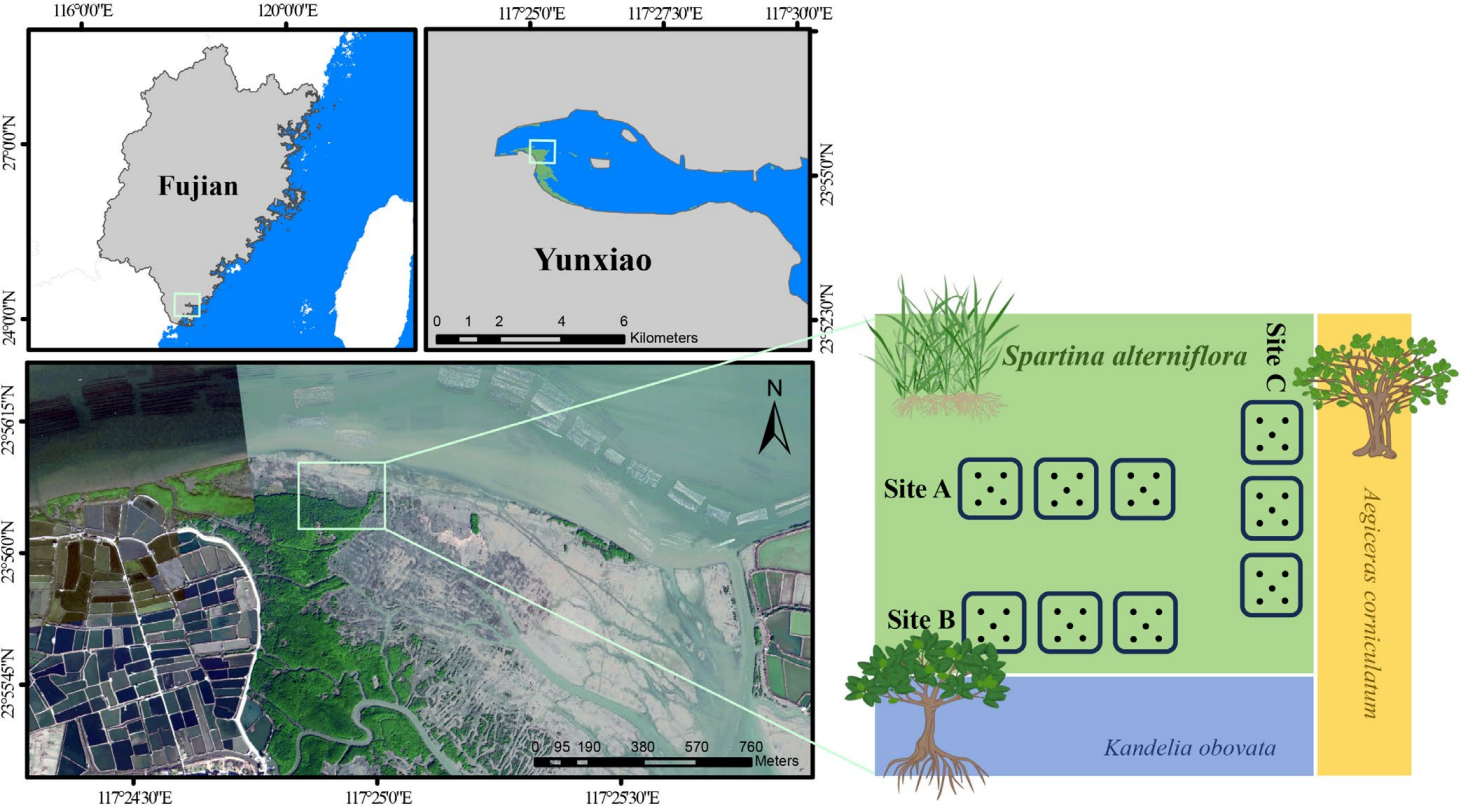
Sampling sites. Soil samples were collected from three sites in Zhangjiang Estuary Mangrove National Nature Reserve in Yunxiao County, Fujian Province, China. As shown in the map, they are Site A (marsh center of *Spartina alterniflora* ), Site B (marsh border of *S. alterniflora* neighboring *Kandelia obovata*), and Site C (marsh border of *S. alterniflora* neighboring *Aegiceras corniculatum*).

In January (senescent period of *S. alterniflora* ) and July (vigorous growth period of *S. alterniflora* ) of 2022, soils were collected, respectively, from three sampling sites in the marsh area of this reserve, including site A (marsh center) occupied only by *S. alterniflora* , site B (marsh border) where *S. alterniflora* neighbors *K. obovata*, and site C (marsh border) where *S. alterniflora* neighbors *A. corniculatum* (Figure 1). Each time, three parallel sample plots (10 × 10 m quadrate) with about 5 m apart were set in every sampling site to avoid microenvironmental autocorrelation. In each plot, the five‐spot sampling method (Zhang, Chen, et al. 2024) was employed and five soil cores (at a depth of 20–30 cm) close to *S. alterniflora* were collected randomly. Soil moisture content was analyzed by placing fresh soil at 60°C until constant weight. Soil temperature was tested in situ, and salinity was measured by pocket refractometer (PAL‐06S, ATAGO, Japan) with a water to air‐dried soil ratio of 5:1 (He et al. 2012). No significant difference was found in these environmental factors (Table S1). Roots of *S. alterniflora* inside these cores were picked out with sterile forceps in the laboratory for rhizosphere soil collection. After the removal of all plant tissues, the remaining soils were mixed completely as a single bulk soil sample. Therefore, three bulk soil samples and three rhizosphere soil samples were collected at each site each time.

Rhizosphere soil samples were collected according to the literature (Bulgarelli et al. 2012; Beckers et al. 2016). Briefly, roots with approximately 1 mm of soil around them were placed in sterile tubes, then sterile phosphate‐buffered saline (PBS) solution was added to wash off the soil from the roots. After removing the roots, the suspension was centrifuged to obtain the rhizosphere soil. Meanwhile, partial soil was scraped directly from the root surface and stored at −20°C for organic acid analysis. All soil samples were subdivided into two parts. One part was stored at −80°C for molecular analysis, and the other part was air‐dried for soil physicochemical analysis. Sample information can be found in Table S1.

### 2.2 Soil Properties and Low‐Molecular‐Weight Organic Acids (LMWOAs) Analysis

The contents of total carbon (TC), total nitrogen (TN) and total sulfur (TS) in the rhizosphere and bulk soils were measured by an organic elemental analyzer (Vario EL Cube, Elementar, Germany). Soil carbon‐nitrogen ratio was calculated using TC and TN. LMWOAs were extracted and detected following the literature with some modifications (Wang et al. 2007; Mimmo et al. 2008). Briefly, water was added to the rhizosphere soil at a water‐to‐soil ratio of 6:1. After full oscillation and centrifugation, the supernatant was collected and freeze‐dried for analysis. Fourteen types of LMWOAs were measured in this study, including oxalic acid, tartaric acid, formic acid, pyruvic acid, malic acid, malonic acid, lactic acid, acetic acid, maleic acid, citric acid, succinic acid, fumaric acid, propanoic acid, and cis‐aconitic acid. They are components of root exudates of various plants (Weng et al. 2013; Seregin and Kozhevnikova 2024) and are closely related to nutrient cycling (Ström et al. 2005; Clarholm et al. 2015; Wang et al. 2015). Their concentrations were quantified with internal standards and determined by high‐performance liquid chromatography (HPLC) using a Thermo Scientific UltiMate 3000 system (Thermo Fisher Scientific, USA). Separation was performed on an Athena C18‐WP column (4.6 × 250 mm, 3 μm) at 30°C. The mobile phase was 20 mM NaH_2_PO_4_ solution, adjusted to pH 2.35, and was delivered at a flow rate of 0.7 mL/min under isocratic elution. UV detection was performed at 210 nm, and all samples and solutions were filtered through a 0.45 μm membrane before use.

### 2.3 DNA Extraction and Amplicon Sequencing

After extraction by DNeasy PowerSoil Pro Kit (Qiagen, Germany), we used agarose gel electrophoresis and Qubit 4 Fluorometer (Thermo Fisher Scientific, USA) to analyze the quality and concentration of soil DNA. Then, we used primers 341F (5′‐CCTAYGGGRBGCASCAG‐3′) and 806R (5′‐GGACTACNNGGGTATCTAAT‐3′) to amplify the V3‐V4 region of bacterial 16S rRNA genes (She et al. 2024), and used primers ITS1F (5′‐CTTGGTCATTTAGAGGAAGTAA‐3′) and ITS2R (5′‐GCTGCGTTCTTCATCGATGC‐3′) to amplify the fungal ITS1‐1F region (Wang et al. 2019). Polymerase chain reaction (PCR) was performed in a Bio‐Rad T100 thermal cycler (Bio‐Rad, USA) using Phusion High‐fidelity PCR Master Mix (New England Biolabs, USA), and the thermal cycling conditions were 98°C for 1 min; 30 cycles of 98°C for 10 s, 50°C for 30 s, and 72°C for 30 s; and a final extension at 72°C for 5 min (Zhang, Chen, et al. 2024). After that, the PCR products were detected by electrophoresis on 2% agarose gel and purified for sequencing. High‐throughput sequencing was performed on the Illumina NovaSeq6000 PE250 platform with a paired‐end protocol by Novogene corporation (Wu et al. 2022). FLASH (http://ccb.jhu.edu/software/FLASH/) was used for sequence assembly (Magoč and Salzberg 2011). Quality control of sequences was performed using fastp software (version 0.23.1) and the UCHIME algorithm (http://www.drive5.com/usearch/manual/uchime_algo.html) (Edgar et al. 2011). We used VSEARCH (version 2.15.0) to remove chimera sequences. Then, denoising was performed by DADA2 algorithm via the QIIME2 software (version 2022.2) to obtain amplicon sequence variants (ASVs) (Callahan et al. 2016), which represent individual sequences with 100% nucleotide identity. ASVs with an absolute abundance less than 5 were filtered out. Silva (version 138.1) and Unite (version 9.0) databases were used for bacterial and fungal ASVs taxonomic annotation, respectively. Prior to downstream analysis, we removed unassigned sequences and rarefied the ASVs based on the sample with the fewest sequences. Totally, 38,626 bacterial and 16,103 fungal ASVs were obtained for each sample. FUNGuildR (version 0.2.0.9000) was used to predict fungal ecological guilds (Nguyen et al. 2016). Only the guild assignments with a confidence ranking of “highly probable” or “probable” were retained.

### 2.4 Metagenomic Sequencing and Processing

Soil samples collected in the vigorous growth period of *S. alterniflora* were selected for metagenomic sequencing using the Illumina NovaSeq X Plus platform (Illumina Inc., San Diego, CA, USA) with a paired‐end protocol (Xiong et al. 2021). Raw data were preprocessed with fastp (https://github.com/OpenGene/fastp). We used Bowtie2 (version 2.4.5) to filter out sequences belonging to the genomes of *K*. *obovata* (RefSeq assembly accession: GCA_021464305.1), *A. corniculatum* (CNGBdb accession number: CNP0001270), and *S. alterniflora* (RefSeq assembly accession: GCA_008808055.3). Then, about 12.9 GB of clean reads were obtained per sample. We used Megahit (version l.0.4‐beta) for clean data assembly, and assembled scaffolds shorter than 500 bp were deleted. Open reading frames (ORFs) were predicted from the scaffolds using MetaGeneMark (version 2.10) (Karlsson et al. 2012). We used CD‐HIT (version 4.5.8) to obtain the non‐redundant gene catalog (Wang et al. 2023), and used Bowtie2 (version 2.4.5) to calculate the number of reads of the genes on each sample alignment. Genes with reads ≤ 2 in each sample were filtered out. The Kyoto Encyclopedia of Genes and Genomes database (KEGG release 110.0) was used for functional annotation. Genes involved in C, N, P, or S metabolism were further recognized based on the reconstruct tool in KEGG Mapper and related literature (Wu et al. 2022; Long et al. 2025).

### 2.5 Statistical Analysis

All statistical analyses were performed by R 4.3.0 and RStudio 1.4. Nonmetric multidimensional scaling (NMDS) was conducted by the “VEGAN” package (Oksanen et al. 2019), as well as the permutational multivariate analysis of variance (PERMANOVA, Adonis), two‐way PERMANOVA, detrended correspondence analysis (DCA), distance‐based redundancy analysis (db‐RDA), canonical correlation analysis (CCA), and Procrustes tests. Richness, Pielou evenness, and Shannon index were used to evaluate microbial alpha diversity. Statistical significance between groups was determined by *t*‐test, one‐way ANOVA, and Tukey HSD test. *p* < 0.05 means statistical significance. Bar plot, stacked bar plot, violin plot, Venn diagram, and heatmap were used for visualization. Linear discriminant analysis effect size (LEfSe) was applied (with Wilcoxon *p*‐value < 0.05, LDA score > 2) to identify bacterial and fungal biomarkers (at genus to phylum levels) of different groups using the package “microeco” (Liu et al. 2021). The top 30 biomarkers with the highest LDA score were displayed each time. The UPGMA cluster analysis was based on “ggtree” package (Yu et al. 2017). The systems theoretic accident model and process (STAMP) was used to identify the differential functional genes (*p* < 0.05) between particular groups based on the *t*‐test (Zhang et al. 2022).

For soil samples collected from each site (12 samples per site), microbial interkingdom network analysis at the ASV level was performed using the “Hmisc” package (Harrell Jr 2024) based on Spearman correlation scores. Sub‐networks of each sample were generated from the original networks, and we used the “igraph” package (Csárdi and Nepusz 2006) to calculate the network topological parameters. Bacterial and fungal ASVs with total relative abundance greater than 0.01% and present in more than 30% of samples were retained for analysis (Zhang, Chen, et al. 2024). Only the strong and significant correlations (Spearman's *r* ≥ 0.8 or r ≤ −0.8; BH‐adjusted *p* < 0.01) were kept. Gephi 0.10 (Bastian et al. 2009) was used for network visualization. Network complexity was reflected by the average degree of nodes. Furthermore, we used cohesion (Herren and McMahon 2017) and robustness (Gao et al. 2022) to assess network stability. For each node, the within‐module connectivity (*Z*_*i*_) and among‐module connectivity (*P*_*i*_) were calculated. Nodes belonging to connectors (*Z*_*i*_ < 2.5; *P*_*i*_ > 0.62), module hubs (*Z*_*i*_ > 2.5; *P*_*i*_ < 0.62), and network hubs (*Z*_*i*_ > 2.5; *P*_*i*_ > 0.62) were identified as keystone taxa of the network (Fan et al. 2018; Guo et al. 2022).

In addition, co‐inertia analysis (CoIA) was used to identify relationships between parallel datasets by package “ade4” (Dray and Dufour 2007). Correlation analyses were applied using the package “psych” (William 2025) and the *lm* function. Meanwhile, a partial least squares path model (PLS‐PM) using the “plspm” package (Sanchez et al. 2024) was also conducted in order to explore the mechanisms by which the rhizosphere effects of *S. alterniflora* respond to interspecific interactions. However, the Goodness‐of‐Fit index (GoF) of the PLS‐PM was only 0.682, which is less than the generally acceptable value (over 0.7). So, PLS‐PM was not adopted.

## 3 Results

### 3.1 Soil Nutrient Properties and Low‐Molecular‐Weight Organic Acids (LMWOAs)

**FIGURE 2:**
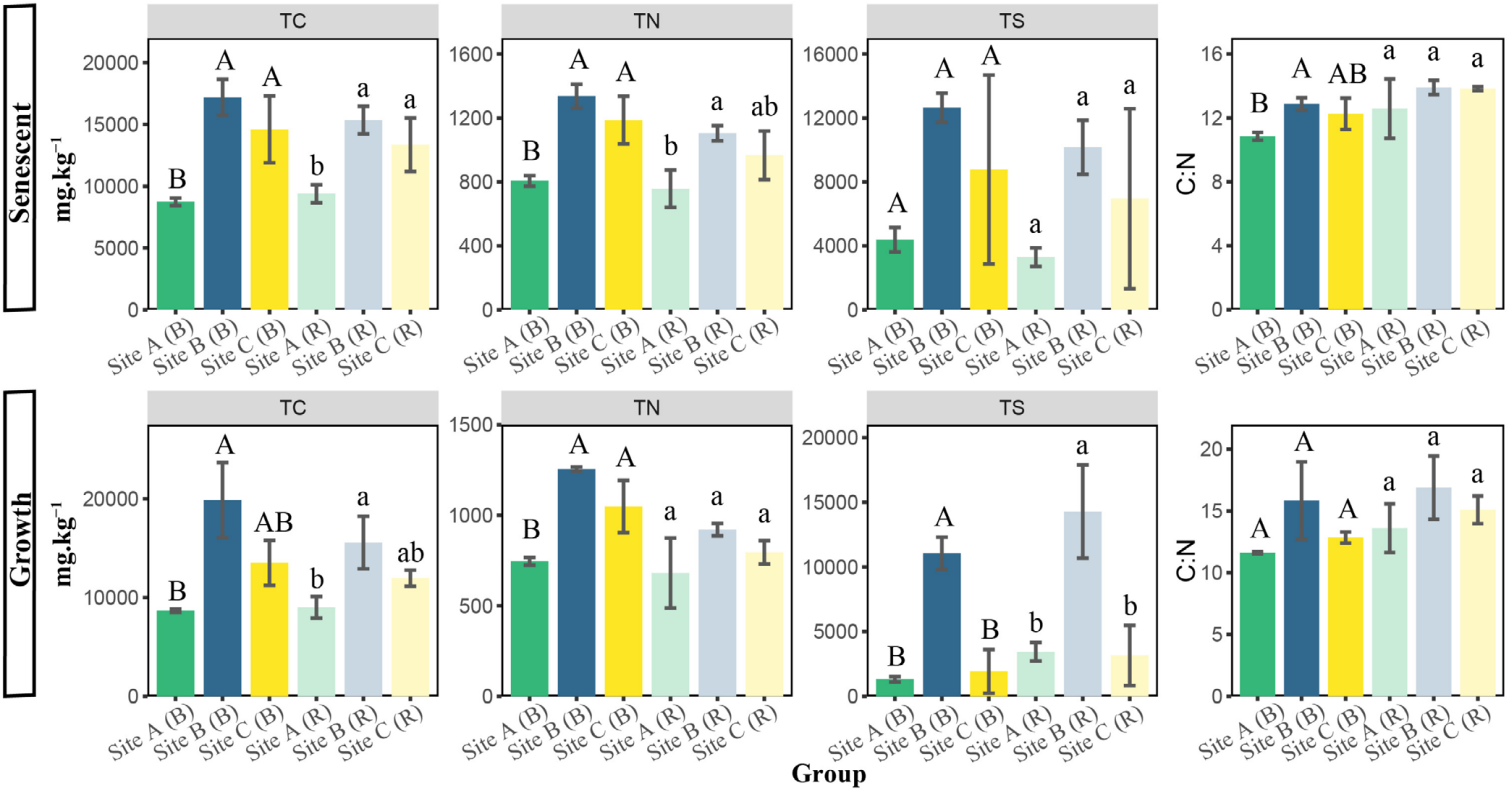
Soil nutrient properties under the influence of plant interspecific interactions. Total carbon (TC), total nitrogen (TN), total sulfur (TS) contents, and carbon–nitrogen ratio (C:N) in the rhizosphere and bulk soils of *S. alterniflora* across different groups. Different groups were colored differently. Different letters indicate the statistical difference between particular groups (*p* < 0.05). Error bars represent standard deviations. Site A: Marsh center of *S. alterniflora* ; Site B: Marsh border of *S. alterniflora* neighboring *K. obovata*; Site C: Marsh border of *S. alterniflora* neighboring *A. corniculatum*. (R): Rhizosphere soil; (B): Bulk soil.

In the senescent period of *Spartina alterniflora* , total carbon (TC) contents in Site B (marsh border of *S. alterniflora* neighboring *Kandelia obovata*) and Site C (marsh border of *S. alterniflora* neighboring *Aegiceras corniculatum*), considering both rhizosphere and bulk soils, were on average 1.81 ± 0.24 and 1.55 ± 0.26 times that of Site A (marsh center of *S. alterniflora* ). Meanwhile, soil total nitrogen (TN) contents in Site B and Site C were on average 1.57 ± 0.22 and 1.39 ± 0.27 times that of Site A (Figure 2). Soil total sulfur (TS) contents were on average 3.06 ± 0.62 times in Site B and 1.93 ± 1.26 times in Site C compared with Site A. In the vigorous growth period, soil TC contents were significantly higher in Site B (15,556.33 ± 2652.38 mg kg^−1^ in rhizosphere and 19,855.67 ± 3806.54 mg kg^−1^ in bulk soil) and Site C (11,963.33 ± 808.65 mg kg^−1^ in rhizosphere and 13,506.33 ± 2278.09 mg kg^−1^ in bulk soil), compared with those in Site A (9008.33 ± 1080.71 mg kg^−1^ in rhizosphere and 8663 ± 171.19 mg kg^−1^ in bulk soil). Almost no significant change in the soil carbon‐nitrogen ratio was observed in the two periods. In addition, no significant variation in nutrient properties between rhizosphere and bulk soils of *S. alterniflora* was found, except for the carbon‐nitrogen ratio in the senescent period and TN content in the vigorous growth period (Figure S1).

**FIGURE 3:**
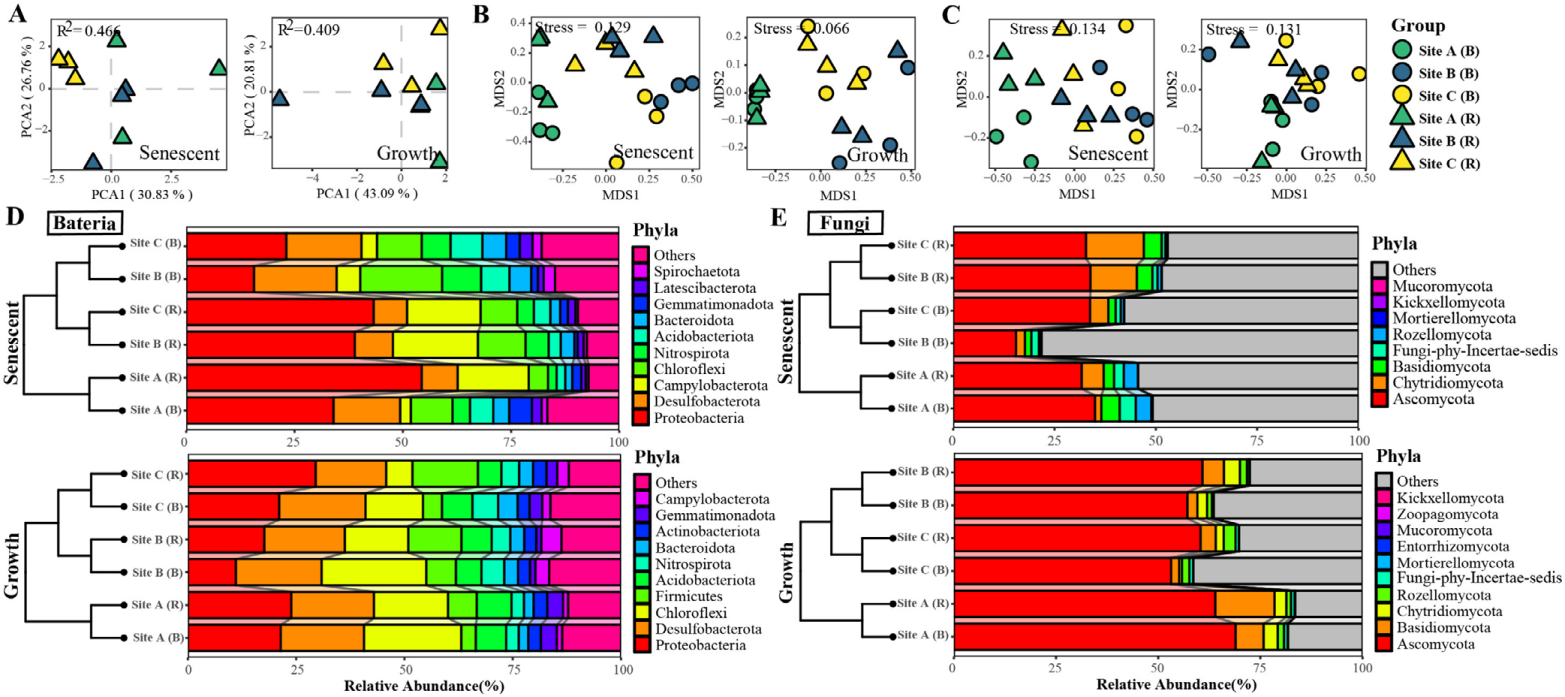
The compositions of low‐molecular‐weight organic acids (LMWOAs) and soil microbial communities changed when *S. alterniflora* was adjacent to mangroves. Principal component analysis (PCA) of LMWOAs in senescent and vigorous growth periods of *S. alterniflora* (A). Nonmetric multidimensional scaling (NMDS) of soil bacterial communities (B) and fungal communities (C) in two periods. Cluster analysis (based on amplicon sequence variants) and community composition at the phylum level of soil bacterial (D) and fungal (E) communities among different groups. Only the top 10 phyla with the highest relative abundance were shown. Site A: Marsh center of *S. alterniflora* ; Site B: Marsh border of *S. alterniflora* neighboring *K. obovata*; Site C: Marsh border of *S. alterniflora* neighboring *A. corniculatum*. (R): Rhizosphere soil; (B): Bulk soil.

Two‐way PERMANOVA showed that LMWOAs were strongly influenced (*p* < 0.05) by both plant phenological stages and interspecific interaction (Table S2). In the senescent period of *S. alterniflora* , LMWOAs of different groups were well separated (*p* < 0.05) with 57.59% of total variation explained by the first two axes of principal component analysis (PCA) (Figure 3A). In the vigorous growth period, the first two axes of PCA can explain 63.90% of total variation of LMWOAs. The contents of LMWOAs in each group are shown in Figure S2, except for oxalic acid, which was present at very low concentrations. Compared to that in Site A (4.77 ± 2.79 μg kg^−1^ soil), the maleic acid in Site B was up to 13.52 ± 1.96 μg kg^−1^ soil, and in Site C it was up to 16.18 ± 5.64 μg kg^−1^ soil in the vigorous growth period. Meanwhile, the malonic acid in Site C was much higher than those of other groups in the senescent period. No significant correlation was observed between LMWOAs and soil nutrient properties (Figure S3).

### 3.2 Composition of Soil Bacterial and Fungal Communities

Significant differences (*p* < 0.05) in the composition of soil bacteria (Figure 3B) and fungi (Figure 3C) among different groups were visualized using nonmetric multidimensional scaling (NMDS) based on Bray–Curtis distances. However, their alpha diversities remained stable (Figure S4). In the senescent period of *S. alterniflora* , only 3.57% bacterial ASVs and 2.40% fungal ASVs in rhizosphere soil were shared among groups, and only 3.84% bacterial ASVs and 1.45% fungal ASVs in bulk soil were shared by Site A, Site B, and Site C. Similarly, only a few bacterial and fungal ASVs were shared among groups in the vigorous growth period (Figure S5). In addition, rhizospheric microorganisms were found to differ significantly from those in bulk soil only in the senescent period (Table S2). Cluster analysis of bacterial and fungal ASVs showed that microbes from Site B and Site C formed a cluster distinct from that of Site A (Figure 3D,E). The most abundant bacterial phyla across all samples were Proteobacteria and Desulfobacterota. In the senescent period, compared with Site A, Chloroflexi, Nitrospirota, and Bacteroidota in Site B and Site C were enriched. Bacteroidota was also increased in Site B and Site C in the vigorous growth period of *S. alterniflora* . For soil fungi, Ascomycota dominated in all samples. In the senescent period of *S. alterniflora* , Chytridiomycota was enriched in Site B and Site C compared to Site A, while Rozellomycota showed the opposite. In the vigorous growth period, Basidiomycota was decreased under the influence of plant interspecific interaction. Furthermore, the saprotrophic fungi dominated in all groups in the two periods (Figure S6). Plant pathogenic fungi in rhizosphere and bulk soils of Site A were more abundant than those in other groups in the senescent period.

Linear discriminant analysis effect size (LEfSe) identified that Nitrospinota, Propionigenium, and other taxa were rhizospheric bacterial biomarkers for Site A in the senescent period (Figure S7A). For Site B, they were Ectothiorhodospirales, Bacteroidota, and so on. Meanwhile, taxa such as Rhizobiales were the rhizospheric biomarkers of Site C. In the bulk soil in Site A, Proteobacteria, Desulfobulbia, and other taxa were identified as the core bacterial taxa. Nitrospirota, Geobacterales, and others were the core microbes in Site B bulk soil, and seven bacterial taxa were also identified as biomarkers of Site C bulk soil. The bacterial biomarkers of different groups in the vigorous growth period of *S. alterniflora* were also identified (Figure S7B). More detailed information on them can be found in Table S3.

In addition, Chaetosphaeriales, Diatrypaceae, and Geastrales were the rhizospheric fungal biomarkers of Site A in the senescent period of *S. alterniflora* (Figure S8A). Meanwhile, Hypocreales and some families of Pleosporales were fungal biomarkers in the bulk soil of Site A. Cystofilobasidiales and Xenoroussoella were the rhizospheric biomarkers of Site C, and Cucurbitinus were the biomarkers in its bulk soil. In the vigorous growth period of *S. alterniflora* , 10 fungal taxa were recognized as core microbes in the Site A rhizosphere (Figure S8B). Meanwhile, Meruliaceae and other taxa were fungal biomarkers in the Site A bulk soil. Rhizophydiomycetes and Nigrogranaceae were identified as rhizosphere biomarkers of Site B. Lulworthiales and others were rhizosphere biomarkers of Site C, and Emericellopsis were biomarkers of its bulk soil.

### 3.3 Microbial Interkingdom Co‐Occurrence Network Analysis

**FIGURE 4:**
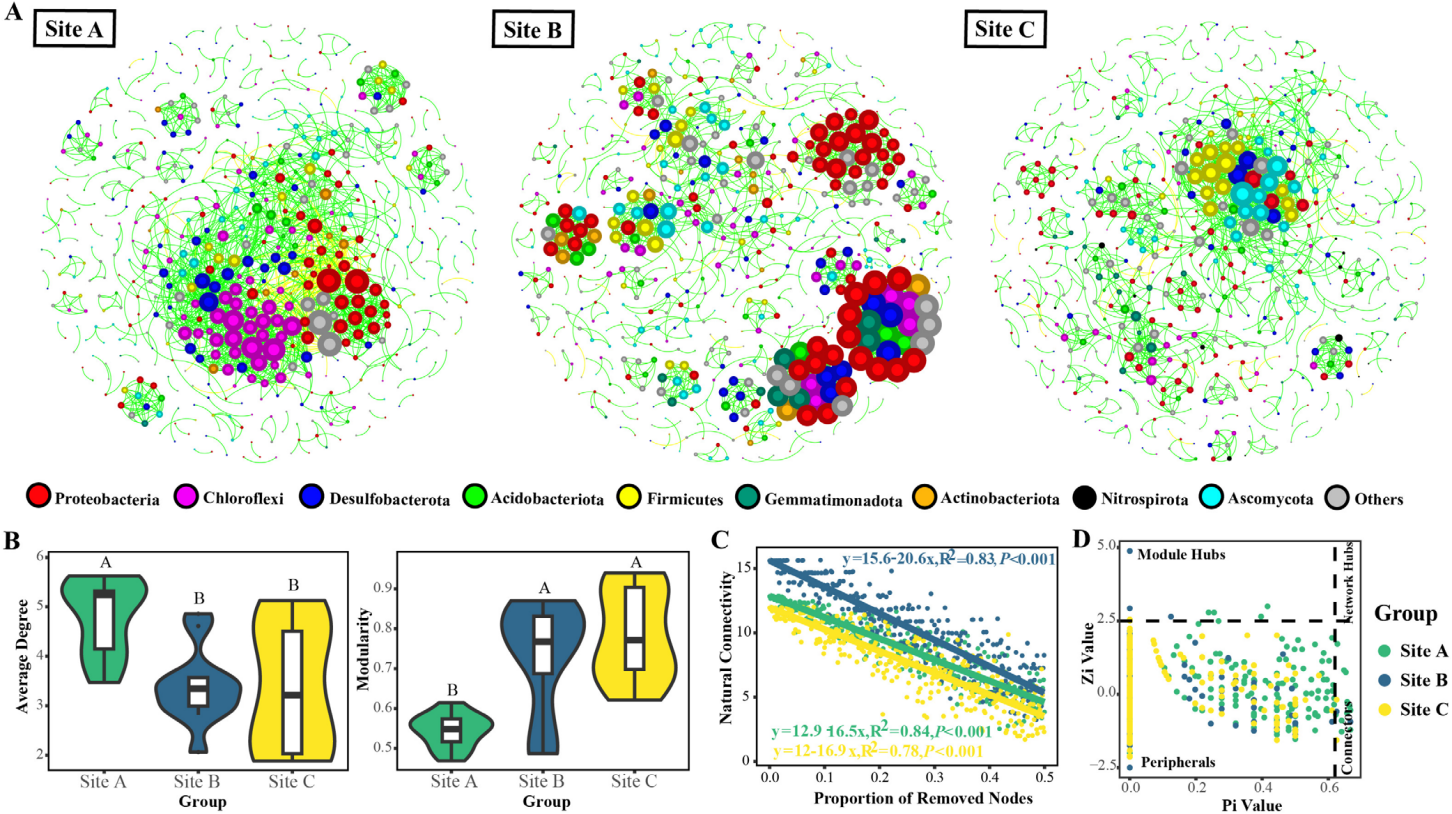
Microbial interkingdom interactions affected by plant interspecific interactions. Microbial interkingdom co‐occurrence networks at the amplicon sequence variants level of different groups (A). The top eight phyla with the most nodes in each network were colored differently. Positive and negative correlations are shown in green and yellow, respectively. Comparison of the average degree of nodes and modularity between different networks (B). Network structural robustness assessed by the degradation of natural connectivity while removing nodes (C). Zi‐Pi plot based on the topological roles of nodes in the networks (D). Site A: Marsh center of *S. alterniflora* ; Site B: Marsh border of *S. alterniflora* neighboring *K. obovata*; Site C: Marsh border of *S. alterniflora* neighboring *A. corniculatum*.

Networks of Site A, Site B, and Site C were composed of 586, 627, and 661 nodes, respectively (Figure 4A). Nodes represent ASVs. Their sizes are proportional to the number of connections (degrees). There were more bacterial nodes than fungal nodes in each network, with 80.89% bacterial nodes in the Site A network, 78.63% and 78.06% bacterial nodes in the Site B and Site C networks, respectively (Table S4). The edge numbers of Site A, Site B, and Site C were 1691, 1652, and 1432, respectively. There were more positive edges in Site B (97.46%) and Site C (97.28%) than in Site A (91.60%). Compared to the Site A network (11.53%), the number of interkingdom edges increased in both the Site B network (23%) and the Site C network (21.51%). Furthermore, the average degree of nodes was significantly lower in the networks of Site B and Site C than in that of Site A (Figure 4B). Higher modularity was also observed in the Site B and Site C networks compared to Site A. In addition, cohesion analyses showed that the ratios of negative–positive cohesion were not significantly different between Site B, Site C, and Site A (Figure S9). Meanwhile, the slopes of the decline of natural connectivity upon node removal from these networks were similar (Figure 4C). Other network topological parameters can be found in Table S4.

Based on Zi‐Pi analysis, 12 bacterial ASVs and three fungal ASVs were identified as keystone species in Site A network (Figure 4D). They are mainly from the bacterial classes Anaerolineae, Desulfobaccia, and Clostridia, and the fungal classes Sordariomycetes and Eurotiomycetes. The bacterial keystones in Site B network belonged to Campylobacteria, Anaerolineae, and Clostridia. Moreover, a taxon from Clostridia was the keystone species of Site C network, together with taxa from Gammaproteobacteria and the fungal classes Dothideomycetes and Sordariomycetes.

### 3.4 The Functional Profiles of Microbial Communities

A total of 8208 genes (KEGG orthology) attributed to 288 KEGG pathway modules were identified in this work. Two‐way PERMANOVA analysis (Table S2) showed that there was no statistical difference between microbial functional genes in rhizosphere and bulk soils of *S. alterniflora* , and hence genes of rhizosphere and bulk soils were considered as replicates here. Microbial functional profiles were notably affected by plant interspecific interaction, as genes (KEGG level4) of Site B, Site C, and Site A belonged to different clusters (Figure S10). Results of two‐way PERMANOVA showed the same (Table S2). In total, 315 genes involved in carbon metabolism, 62 genes involved in nitrogen metabolism, 33 genes involved in phosphorus metabolism, and 92 genes involved in sulfur metabolism were further identified. Their dissimilarities in different groups were visualized by NMDS based on Bray–Curtis distances (Figure S11A). There was no significant difference in the standardized absolute abundance of these nutrient cycling genes among different groups, except that the abundance of C metabolism‐related genes in Site B rhizosphere was much higher (*p* < 0.05) than that in other groups (Figure S11B).

**FIGURE 5:**
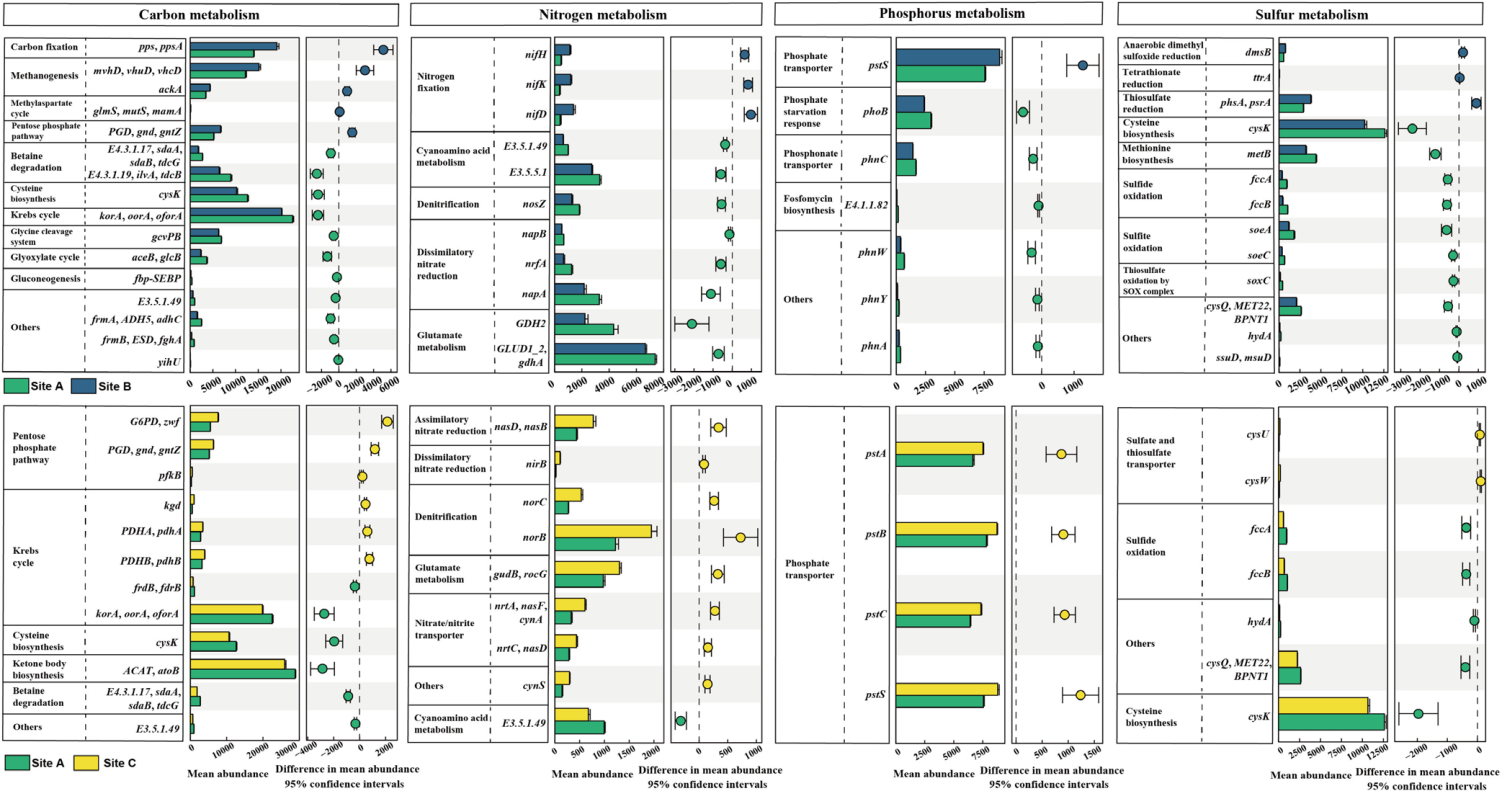
Microbial functional genes involved in C, N, P, or S metabolism were compared between Site B and Site A, and between Site C and Site A. Only the genes with significant differences (*p* < 0.05) between groups were shown. Some differential functional genes involved in C, N, P, or S metabolism that could not be precisely assigned to a specific module or pathway were classified as “Others”. Error bars represent 95% confidence intervals. Site A: Marsh center of *S. alterniflora* ; Site B: Marsh border of *S. alterniflora* neighboring *K. obovata*; Site C: Marsh border of *S. alterniflora* neighboring *A. corniculatum*.

For C metabolism, the standardized absolute abundances of 11 genes in Site B and six genes in Site C were reduced compared to those of Site A (Figure 5). Among them, some Krebs cycle‐related genes (*korA*, *oorA*, *oforA*) and other genes were depleted in both groups. On the contrary, pentose phosphate cycle‐related genes (*PGD*, *gnd*, *gntZ*) significantly enriched in both Site B and Site C. In Site B, few genes involved in methanogenesis (*ackA*, *mvhD*, etc.) and carbon fixation (*pps*, *ppsA*) were also significantly enriched. For N metabolism, Site B has a higher abundance of genes involved in nitrogen fixation (*nifH*, *1nifK*, etc.) than Site A. Genes related to nitrate reduction (*nirB*, *nasD*, *nasB*) and denitrification (*norC*, *norB*) also significantly enriched in Site C compared to those in Site A. However, genes involved in dissimilatory nitrate reduction (*napA*, *nrfA*, *napB*) and denitrification (*nosZ*) were inhibited in Site B.

Compared to Site A, genes of phosphate transporter (*pstA*, *pstB*, *pstC*, *pstS*) were notably enriched in Site C. Among them, gene *pstS* was also enriched in Site B, while the other six genes related to P metabolism were inhibited in Site B, such as gene *phoB*, which is related to the phosphate starvation response. For S metabolism, abundances of *cysK*, *cysQ*, *fccB*, *fccA*, and other genes were significantly decreased in both Site B and Site C compared to those of Site A. The gene *cysK* was involved in cysteine and methionine metabolism, while *fccA* and *fccB* were involved in sulfide oxidation. In addition, *ttrA*, *phsA*, *dmsB*, and *ttrA* were enriched in Site B, and *cysU*, *cysW* were enriched in Site C. More detailed information on these differential genes can be found in Table S5.

### 3.5 Relationships of Soil Properties, LMWOAs, and Microbial Taxonomic and Functional Profiles

**FIGURE 6:**
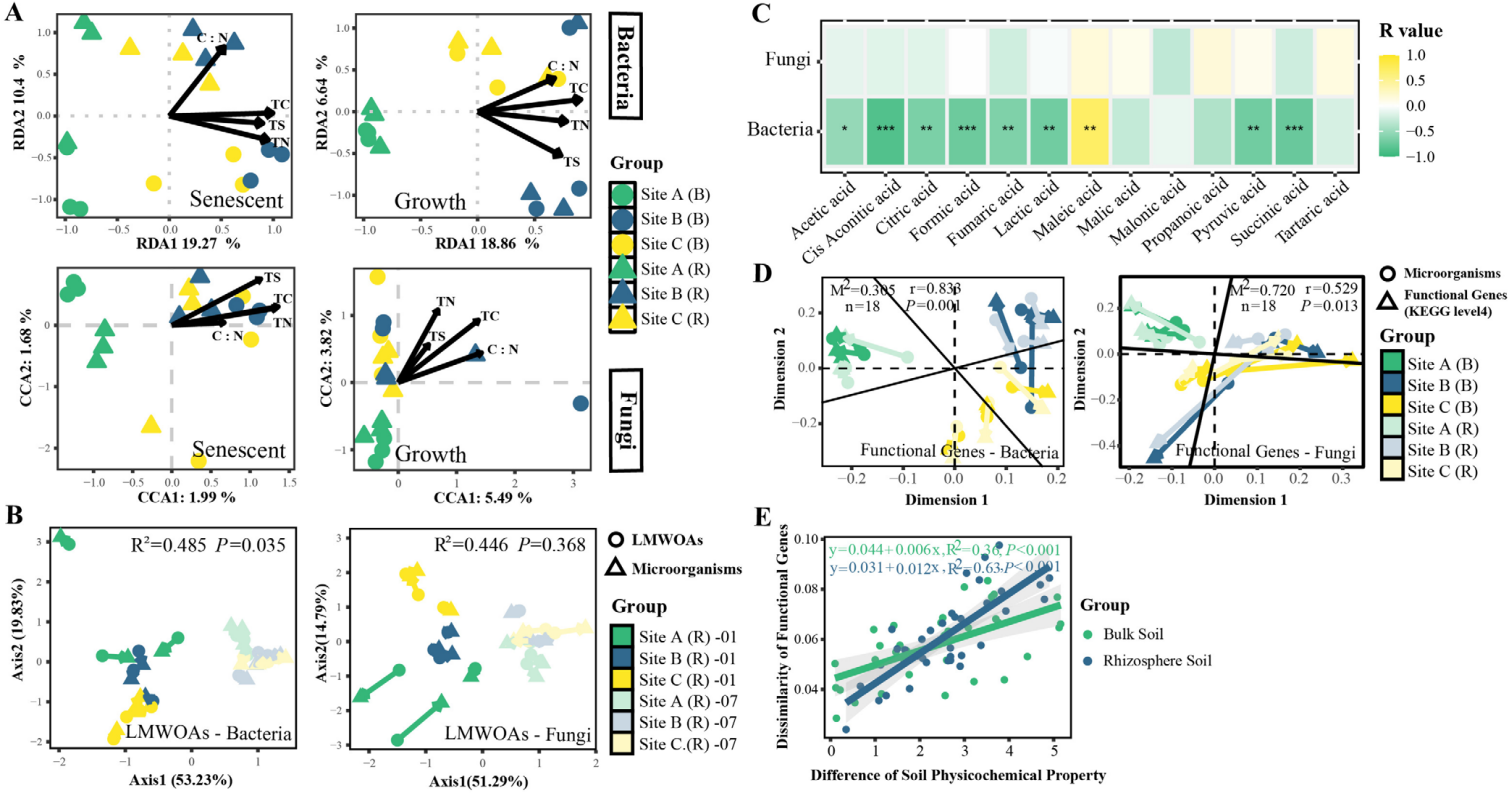
Underlying mechanisms of belowground responses of *S. alterniflora* to plant interspecific interactions. Relationships between soil nutrient properties and the bacterial and fungal communities (A). C:N, Carbon–nitrogen ratio; TC, total carbon; TN, total nitrogen; TS, total sulfur. Co‐inertia analysis between LMWOAs and soil microbiomes (B). Heat map showing the Spearman correlations between different types of LMWOAs and soil bacteria and fungi (C). “*” means *p* < 0.05; “**” means 0.001 < *p* < 0.01; “***” means *p* < 0.001. Procrustes analysis between functional genes (KEGG level4) and soil microbiomes (D). Correlations between soil nutrient properties and KEGG level4 functional genes (E). Site A: Marsh center of *S. alterniflora* ; Site B: Marsh border of *S. alterniflora* neighboring *K. obovata*; Site C: Marsh border of *S. alterniflora* neighboring *A. corniculatum*. (R): Rhizosphere soil; (B): Bulk soil. 01: Senescent period of *S. alterniflora* ; 07: Vigorous growth period of *S. alterniflora* .

Considering the results of detrended correspondence analysis (DCA, max axis lengths: 3.89 and 3.23), relationships between soil properties and bacterial communities were investigated by distance‐based redundancy analysis (db‐RDA) (Figure 6A). Meanwhile, canonical correlation analysis (CCA) was performed on fungal communities (DCA, max axis lengths 4.95 and 4.58). Results showed that soil nutrient properties, including TC, TN, TS, and carbon‐nitrogen ratio, could explain 29.67% and 25.5% of the total variation in bacterial communities in the senescent and vigorous growth periods of *S. alterniflora* , respectively. Only 3.67% and 9.31% of the variation in fungal communities were explained by the first two axes of CCA in these two periods. Co‐inertia analysis (CoIA) indicated that there was a significant co‐variation between LMWOAs and *S. alterniflora* rhizosphere soil bacterial communities, but no association was observed between LMWOAs and fungal communities (Figure 6B). Among these LMWOAs, maleic acid was found to be strongly (*p* < 0.05) and positively associated with soil bacteria according to Spearman correlation analysis, while cis‐aconitic acid, formic acid, and other acids showed opposite correlations (Figure 6C). In addition, a significant correlation between soil bacteria and fungi was recognized (Figure S12). Procrustes analysis using Bray–Curtis dissimilarity metric showed that changes in functional genes were highly correlated (*p* < 0.05) with the compositional shifts of both bacterial and fungal communities (Figure 6D). Meanwhile, functional genes were also significantly and positively correlated with soil nutrient properties in both rhizosphere and bulk soils (Figure 6E).

## 4 Discussion

### 4.1 Soil Nutrients and LMWOAs Responding Belowground Plant Interaction

High resource capture ability and resource utilization efficiency are important biological bases of successful invasive plants (Shen et al. 2011; Sardans et al. 2017). In this study, soil total carbon (TC) and total nitrogen (TN) significantly increased when *S. alterniflora* was adjacent to mangroves, likely attributable to its well‐developed root system and plant–soil–microbe feedback during (Yang et al. 2016; Feng et al. 2017). This is consistent with Wang et al. (2016), which showed that the soil nutrient status was improved in mangrove–Spartina ecotones compared to *S. alterniflora* marsh. Root exudates can help plants with defense and environmental adaptation (Dakora and Phillips 2002; Baetz and Martinoia 2014). LMWOAs are sensitive to environmental conditions. Those released by *S. alterniflora* neighboring mangroves shifted notably, especially maleic acid and malonic acid, which can effectively inhibit plant pathogenic fungi (Fei et al. 2023). They may play important roles in enhancing the belowground competitive ability of *S. alterniflora* neighboring mangroves.

### 4.2 Variations of Microbial Composition and Interaction Patterns

Plant interspecific interaction significantly reshaped the composition of the soil microbiome of *S. alterniflora* in this work. Proteobacteria were reported as the most abundant bacteria in the *S. alterniflora* salt marsh (Gao et al. 2022), and Desulfobacterota commonly occur in ecosystems with sulfate reduction characteristics (Wen et al. 2022). They were the dominant bacterial phyla in all samples in this work. When *S. alterniflora* was adjacent to mangroves, the relative abundance of Bacteroidota increased in both rhizosphere and bulk soils, and some taxa of Chloroflexi were identified as rhizospheric bacterial biomarkers of *S. alterniflora* . This might explain why soil TC and TN contents were higher in sites where *S. alterniflora* neighbored mangroves, as Bacteroidota actively participate in global nutrient cycling (Pan et al. 2023). Meanwhile, Chloroflexi are involved in photoautotrophic carbon fixation (Bovio‐Winkler et al. 2023). In addition, taxa from Nitrospinia were also biomarkers in the *S. alterniflora* marsh. This has confirmed the remarkable ability that *S. alterniflora* has on regulating soil nitrogen (Gao et al. 2019), as most Nitrospinota are nitrite‐oxidizing bacteria (Kop et al. 2024).

Soil fungi are essential for the disease suppression and environmental stress tolerance of plants (Bollmann‐Giolai et al. 2022). Their composition also significantly shifted during *S. alterniflora* invasion (Yang et al. 2019). Plant pathogenic fungi decreased when *S. alterniflora* was adjacent to mangroves. This phenomenon also existed in Chinese Yellow‐River‐Delta National Nature Reserve (Zhang et al. 2021). In addition, taxa from Lulworthiales were the rhizospheric fungal biomarkers of *S. alterniflora* neighboring *A. corniculatum*. They may help with nutrient recycling by decomposing complex lignocellulose compounds (Raghukumar 2017). Furthermore, mycotoxin‐producing Stachybotrys was one of the biomarkers in the marsh center of *S. alterniflora* (Fung et al. 1998). However, as our understanding of the functions of different fungal taxa remains limited, it is unclear what roles these biomarkers actually played in different groups.

Bacteria and fungi can interact in several ways (Peleg et al. 2010; Steffan et al. 2020). In this study, plant interspecific interaction when *S. alterniflora* neighbored mangroves strengthened the cross‐kingdom links between bacteria and fungi, whereas it weakened the within‐kingdom interactions, leading to simpler and looser microbial networks (Zhang, Chen, et al. 2024). Although network structure stability was supported by its complexity (Zhang, Lei, et al. 2024), the stability of *S. alterniflora* soil microbial communities did not significantly decline under plant interspecific interaction in the present work. Moreover, modularity analysis suggests that there was a stronger niche differentiation of the soil microbiome when *S. alterniflora* was adjacent to mangroves, which may benefit plant growth and stress tolerance (Beckers et al. 2017; Zhang et al. 2020). A study on *Amaranthus palmeri* also found that invasive plants can promote a more effective and stable microbial network by reducing the average degree while increasing modularity (Zhang et al. 2023). Zi‐Pi analysis showed that the dominant microbial taxa in *S. alterniflora* soil, such as Proteobacteria and Desulfobacterota, also played important roles in the interkingdom co‐occurrence networks, highlighting their essential functions in wetland ecosystems (An et al. 2019; Wang et al. 2025).

### 4.3 Changes of Microbial Functional Genes Responding Plant Interaction

Microbial functional genes suggested the potential changes in soil nutrient cycling when *S. alterniflora* grew adjacent to mangroves. Due to the functional redundancy of microorganisms (Louca et al. 2018), no significant variation of functional genes was found between the rhizosphere and bulk soils of *S. alterniflora* . For soil carbon metabolism, previous studies reported that *S. alterniflora* increased the methane emissions in blue carbon ecosystems via alteration of methane cycling bacteria and archaea (Yuan, Ding, et al. 2014; Bu et al. 2018). In this work, microbial functional genes related to methanogenesis and carbon fixation were notably enriched in sites where *S. alterniflora* neared mangroves, indicating a potential increase in CH_4_ emissions (Wang et al. 2024). Enhanced carbon input provides the basis for the generation of greenhouse gases such as methane (Cheng et al. 2007). The alterations in soil structure and properties caused by the roots of *S. alterniflora* can also contribute to the increase in greenhouse gas emissions (Li et al. 2024).

Based on isotope technology and gas chromatography, Gao et al. (2019) revealed that wetland N_2_O emissions were enhanced by *S. alterniflora* , with stimulation of soil denitrification. Similarly, Chen et al. (2023) demonstrated that both nitrogen fixation and removal processes were facilitated by *S. alterniflora* . In line with these findings, functional genes involved in nitrogen fixation were notably enriched when *S. alterniflora* was neighboring mangroves in this study. Whereas nitrate reduction and denitrification processes were strengthened when *S. alterniflora* neighbored *A. corniculatum*, they were weakened when it neighbored *K. obovata*. This indicated that *S. alterniflora* may have different responsive patterns in nitrogen metabolism when competing with *K. obovata* and *A. corniculatum*.

A strong coupled relationship between sulfur metabolism and nitrogen fixation existed in *S. alterniflora* roots (Rolando et al. 2024). It has been reported that *S. alterniflora* root systems can catalyze sulfide oxidation (Lee et al. 1999). However, this capability appears to be weakened as the abundance of genes involved in sulfide and sulfite oxidation decreased under the influence of plant interspecific interactions (Lü et al. 2017). Moreover, enriched *phsA*, *cysU*, and related genes indicated that microbial anaerobic respiration was accelerated when *S. alterniflora* neighbored *K. obovata* (Burns and DiChristina 2009), and sulfate transport was enhanced when it neighbored *A. corniculatum* (Goff and Yee 2017).

In addition, *S. alterniflora* invasion will promote soil phosphate availability, lower the soil phosphorus pool stability and alter soil phosphate‐solubilizing bacteria (Lin et al. 2024; Zhang, Yang, et al. 2024). Here, metagenomic results suggested that the abundance of genes related to phosphate transporters was significantly enhanced when *S. alterniflora* was adjacent to mangroves. This may alleviate the phosphorus limitation of *S. alterniflora* to some extent and enhance its competitive ability, since mangroves appear to face a stronger phosphorus limitation (McKee et al. 2002; Feller et al. 2003). However, it is important to note that there are likely other genes and metabolic pathways that have not been detected. Thus, it is impossible to make a definitive conclusion about how plant interspecific interactions influence soil nutrient cycling.

### 4.4 Rhizosphere Effect in Belowground Interspecific Interaction

The rhizosphere effect plays an important role in belowground competition through plant–soil–microbe interactions (Bais et al. 2004; Hodge and Fitter 2013; Guerrieri and Rasmann 2024). Microbial communities can be influenced by soil nutrient conditions (Islam et al. 2020). For instance, a study of terrestrial ecosystems indicated that soil carbon, nitrogen, and their stoichiometric ratios were significantly correlated with microbial community structures (Gaudel et al. 2023). Meanwhile, sulfur application stimulated the malic acid secretion by soybean roots, which further affected soil bacterial communities (Damo et al. 2023). In this work, significant correlations between microorganisms and soil nutrient properties were found. However, the total variation of microbial communities explained by the selected environmental variables was limited. This may be due to the limited number of tested soil properties. LMWOAs were associated with the variation in rhizosphere bacteria of *S. alterniflora* in this study, although not all types of acids tested showed significant associations. Studies on invasive plants, like *Ambrosia trifida* , also displayed that root exudates can modulate rhizospheric microbiome colonization (Yuan, Tang, et al. 2014; Li et al. 2022), while the changes in soil microorganisms may enhance plants' adaptation to the environment and alleviate their pressure on resource acquisition (Hamilton and Frank 2001; Lau and Lennon 2012). These results suggest that the variations of rhizospheric LMWOAs and soil microorganisms of *S. alterniflora* neighboring mangroves may intensify its competitiveness. This has also been observed in other invasive plants, such as the indirect competition mediated by soil communities that has contributed to the invasion success of *Holcus lanatus* in California coastal prairies (Bennett et al. 2011), while *Ligustrum sinense* and other species benefited from the alterations in arbuscular mycorrhizal fungi during invasion (Greipsson and DiTommaso 2006).

Network analysis is widely used to infer microbial interactions, although correlations do not necessarily reflect direct biological interactions (Matchado et al. 2021; Srinivasan et al. 2024). A great simplification of microbial networks can result in the reduction of stability (Luo et al. 2023; Zhang, Lei, et al. 2024), but the general assertion that higher complexity universally implies higher stability is oversimplified and requires contextual consideration (Guseva et al. 2022). Meanwhile, higher modularity can contribute to network stability, as it can limit the spread of negative interference (Grilli et al. 2016; Gilarranz et al. 2017). A trade‐off between microbial network complexity and stability exists (Yonatan et al. 2022; Ye et al. 2023). Here, accompanying the significant changes in network complexity and modularity, the microbial community stability of *S. alterniflora* did not decline significantly under the pressure from neighboring mangroves. This might demonstrate the intensified competition of *S. alterniflora* in microbial community stability.

Along with the alterations of soil properties and microorganisms, shifts in functional genes related to nutrient cycling were also found in the present work. The alteration of soil nutrient cycling processes can also facilitate plant invasion (Ahmad Dar et al. 2023; Negesse et al. 2025). For example, a higher nitrogen cycling rate was observed in the invasive *Mikania micrantha* rhizosphere, which provided it with greater competitiveness in obtaining available nitrogen compared to native competitors (Yu et al. 2020). Moreover, accelerated decomposition of soil organic phosphorus contributed to the rapid expansion of invasive species in various environments (Chapuis‐Lardy et al. 2006; Sun et al. 2022). In this study, changes in functional genes related to core nutrient cycling (carbon, nitrogen, phosphate, and sulfur) revealed the adjustment of metabolic mechanisms of *S. alterniflora* neighboring mangroves, which may enhance its competitiveness. However, as this study investigated limited samples at Zhangjiang Estuary mangrove wetland, further studies based on more samples at larger temporal and spatial scales are needed to validate the universality of its findings.

## 5 Conclusions

This work demonstrated the rhizospheric response of the invasive *Spartina alterniflora* adjacent to mangroves through a field investigation. Soil nutrient status was improved in both vigorous growth and senescent periods. Meanwhile, low‐molecular‐weight organic acids (LMWOAs) in its rhizosphere showed significant changes, and strong correlations were detected between LMWOAs and the rhizospheric bacteriome of *S. alterniflora* . Compositional shifts of bacterial and fungal communities in the rhizosphere and bulk soils of *S. alterniflora* adjacent to mangroves were also observed. Moreover, microbial co‐occurrence networks became simpler, accompanied by the increase of network modularity. Microbial functional genes were also significantly affected by plant interspecific interaction, especially genes involved in C, N, P, and S metabolisms. In conclusion, this work revealed the belowground interspecific interaction of the invasive *S. alterniflora* with neighboring mangroves from multiple aspects of rhizosphere effect. These findings advance our fundamental understanding of the invasion strategies employed by *S. alterniflora* in the Zhangjiang Estuary wetland.

## Author Contributions

**Dandan Long:** formal analysis (lead), investigation (equal), visualization (lead), writing – original draft (lead). **Wentao Zhao:** investigation (equal). **Xishuai Li:** investigation (equal). **Qing Sun:** investigation (equal). **Jiqiu Li:** writing – review and editing (lead). **Xiaofeng Lin:** conceptualization (lead), methodology (lead), supervision (lead).

## Funding

This study was supported by the National Natural Science Foundation of China (42176145 and 42076113) and the Fundamental Research Funds for the Central Universities (20720200106 and 20720200109).

## Conflicts of Interest

The authors declare no conflicts of interest.

## Supporting information


**Figure S1:** Comparison of nutrient between rhizosphere and bulk soils of *S. alterniflora*.
**Figure S2:** Bar plot exhibited the contents of different types of low molecular‐weight organic acids in the rhizosphere soil of *S. alterniflora* in different groups.
**Figure S3:** Linear regression and co‐inertia analysis between LMWOAs and rhizosphere soil properties.
**Figure S4:** Alpha diversities of soil bacterial (A) and fungal (B) communities between different groups.
**Figure S5:** Venn diagrams showing overlapped bacterial (A) and fungal (B) ASVs in rhizosphere and bulk soils among different groups in senescent and growth periods of *S. alterniflora*.
**Figure S6:** Functional guild composition of fungi in rhizosphere and bulk soils of *S. alterniflora* in different groups.
**Figure S7:** Bacterial biomarkers in rhizosphere and bulk soils of *S. alterniflora* in different groups were identified via linear discriminant analysis effect size (LEfSe) analysis in senescent (A) and vigorous growth (B) periods.
**Figure S8:** Fungal biomarkers in rhizosphere and bulk soil of *S. alterniflora* in different groups were identified via LEfSe analysis in senescent (A) and vigorous growth (B) periods.
**Figure S9:** Absolute value of the ratio of negative: positive cohesion of different groups.
**Figure S10:** Cluster analysis basing on total functional genes (KEGG level4).
**Figure S11:** The variation of microbial functional genes induced by plant interspecific interactions.
**Figure S12:** Linear relationship between soil bacterial and fungal communities.

**Table S1:** Sampling sites and sample information.
**Table S2:** Results of two‐way Permutational Multivariate Analysis of Variance (two‐way Adonis) in this work.
**Table S3:** Microbial biomarkers recognized in rhizosphere and bulk soils of *S. alterniflora* in different groups.
**Table S4:** Key characteristics of microbial interkingdom co‐occurrence networks of different groups.
**Table S5:** Significantly changed functional genes related to C, N, P, and S metabolisms identified by systems theoretic accident model and process (STAMP).

## Data Availability

Amplicon and metagenomic raw sequence data are available in the NCBI Sequence Read Archive (SRA) with accession numbers PRJNA1231030 and PRJNA1232220, respectively. Further inquiries can be directed to the corresponding author.
