# Supplementary material for: Rhizosphere Effect Enhances Belowground Competition of Coastal Invasive Spartina alterniflora With Mangroves
Source: Ecol Evol. 2025 Nov 30;15(12):e72565. doi: 10.1002/ece3.72565 (PMC12665386; doi:10.1002/ece3.72565)
Supplement: Supplementary file 1 — Figure S1: Comparison of nutrient between rhizosphere and bulk soils of S. alterniflora. Figure S2: Bar plot exhibited the contents of different types of low molecular‐weight organic acids in the rhizosphere soil of S. alterniflora in different groups. Figure S3: Linear regression and co‐inertia analysis between LMWOAs and rhizosphere soil properties. Figure S4: Alpha diversities of soil bacterial (A) and fungal (B) communities between different groups. Figure S5: Venn diagrams showing overlapped bacterial (A) and fungal (B) ASVs in rhizosphere and bulk soils among different groups in senescent and growth periods of S. alterniflora. Figure S6: Functional guild composition of fungi in rhizosphere and bulk soils of S. alterniflora in different groups. Figure S7: Bacterial biomarkers in rhizosphere and bulk soils of S. alterniflora in different groups were identified via linear discriminant analysis effect size (LEfSe) analysis in senescent (A) and vigorous growth (B) periods. Figure S8: Fungal biomarkers in rhizosphere and bulk soil of S. alterniflora in different groups were identified via LEfSe analysis in senescent (A) and vigorous growth (B) periods. Figure S9: Absolute value of the ratio of negative: positive cohesion of different groups. Figure S10: Cluster analysis basing on total functional genes (KEGG level4). Figure S11: The variation of microbial functional genes induced by plant interspecific interactions. Figure S12: Linear relationship between soil bacterial and fungal communities. [file ECE3-15-e72565-s001.docx]

**Rhizosphere effect enhances belowground competition of coastal invasive *Spartina alterniflora* with mangroves**

Dandan Long^1^, Wentao Zhao^1^, Xishuai Li^1^, Qing Sun^1^, Jiqiu Li^1,2^, Xiaofeng Lin^1,2,*^

^1^ Key Laboratory of Ministry of Education for Coastal and Wetland Ecosystems, Fujian Province Key Laboratory for Coastal Ecology and Environmental Studies, College of the Environment and Ecology, Xiamen University, Xiamen 361102, China;

^2^ State Key Laboratory of Marine Environmental Science, National Observation and Research Station for the Taiwan Strait Marine Ecosystem, Xiamen University, Zhangzhou, China, Xiamen University, Xiamen 361102, China.

^*^ Corresponding author:

Xiaofeng Lin, linxf@xmu.edu.cn

10 Pages, 12 Figures


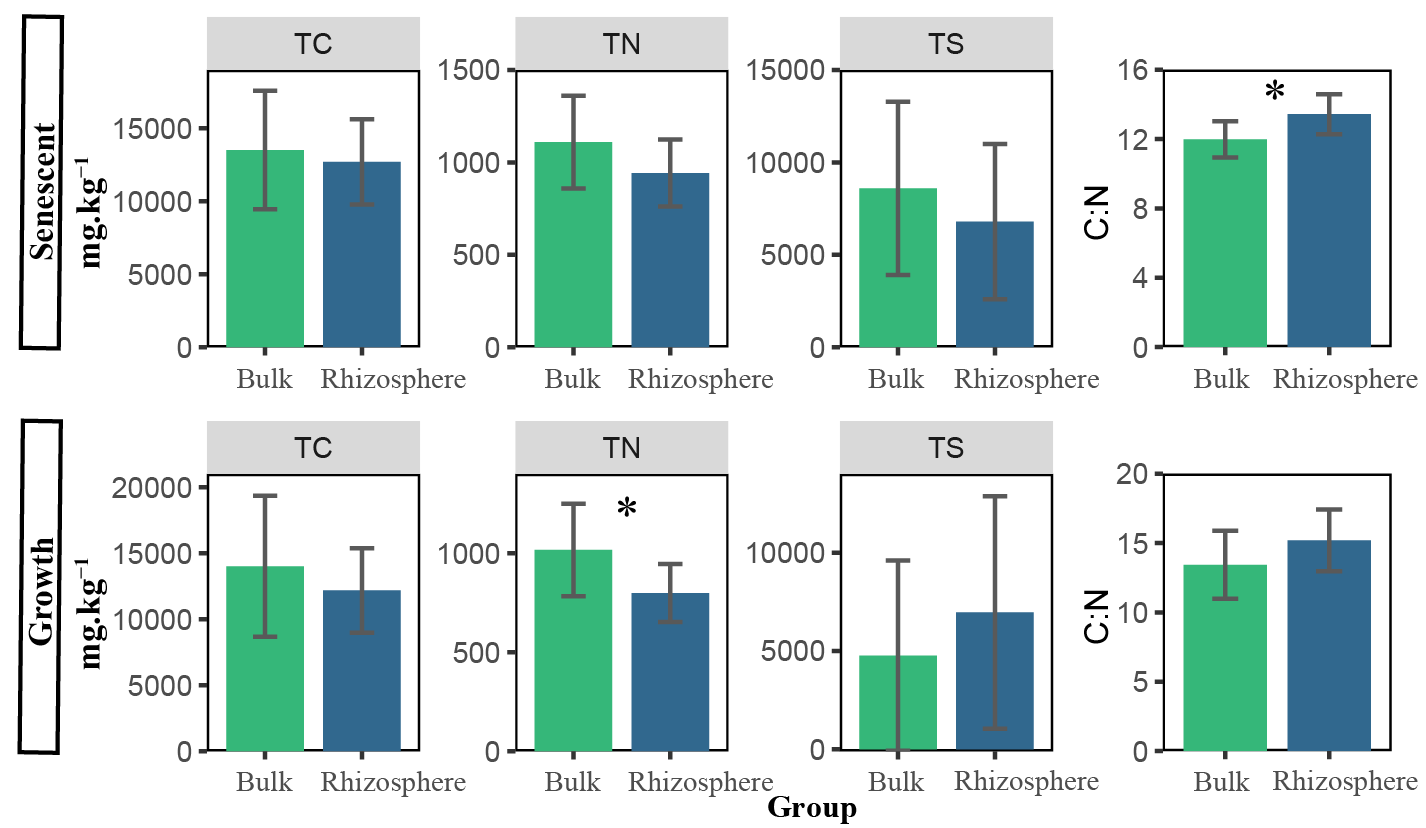


**Fig. S1** **Comparison of nutrient between rhizosphere and bulk soils of *S. alterniflora*.** Soil total carbon (TC), total nitrogen (TN), total sulfur (TS) content, and carbon-nitrogen ratio (C:N) in two different seasons. “*” means *P* < 0.05.


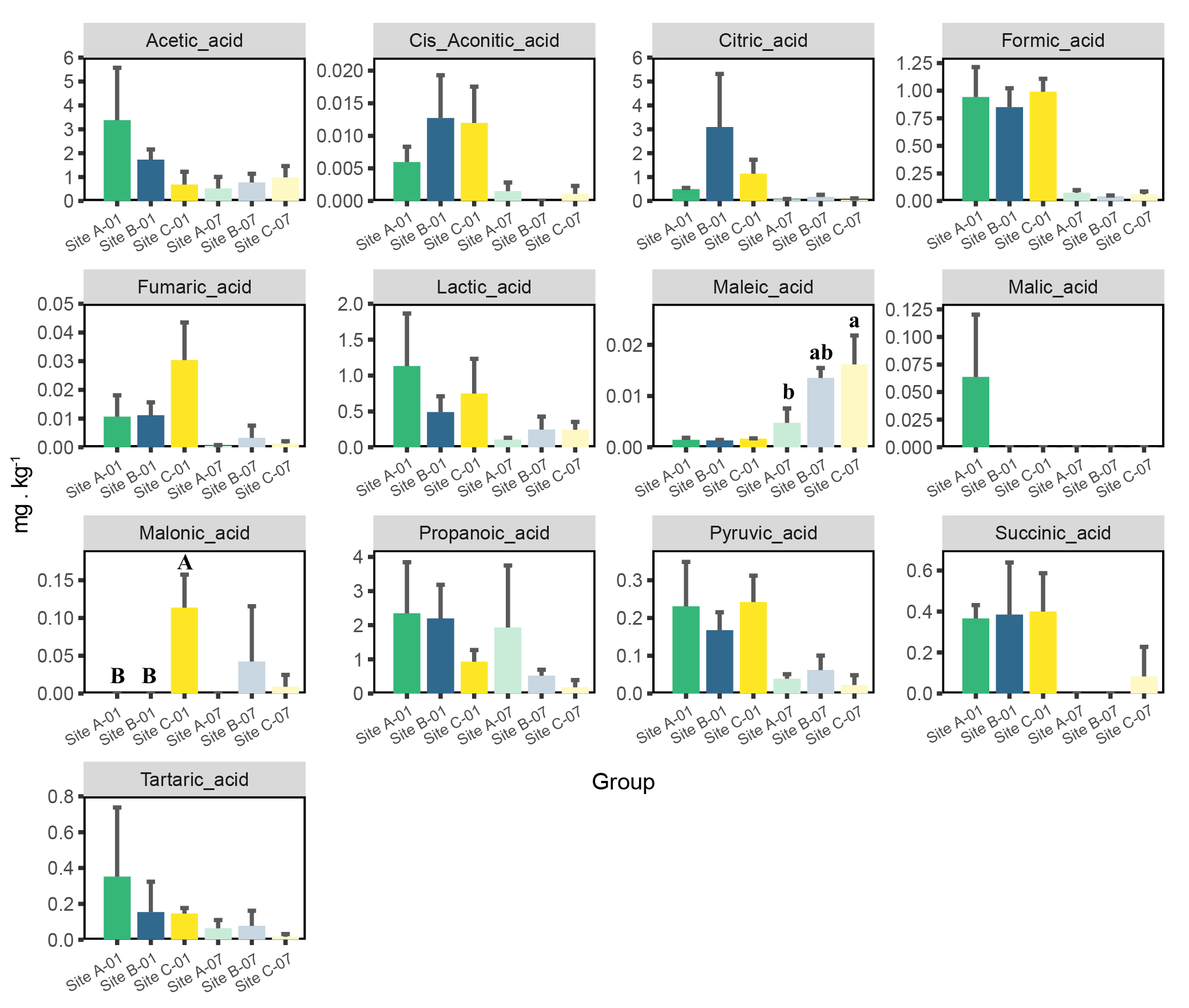


**Fig. S2** **Bar plot exhibited the contents of different types of low molecular-weight organic acids in the rhizosphere soil of *S. alterniflora* in different groups.** Different letters indicate the statistical difference between particular groups (*P* < 0.05). Site A: marsh center of *S. alterniflora*; Site B: marsh border of *S. alterniflora* neighboring *K. obovata*; Site C: marsh border of *S. alterniflora* neighboring *A. corniculatum*. 01: senescent period of *S. alterniflora*; 07: vigorous growth period of *S. alterniflora*.


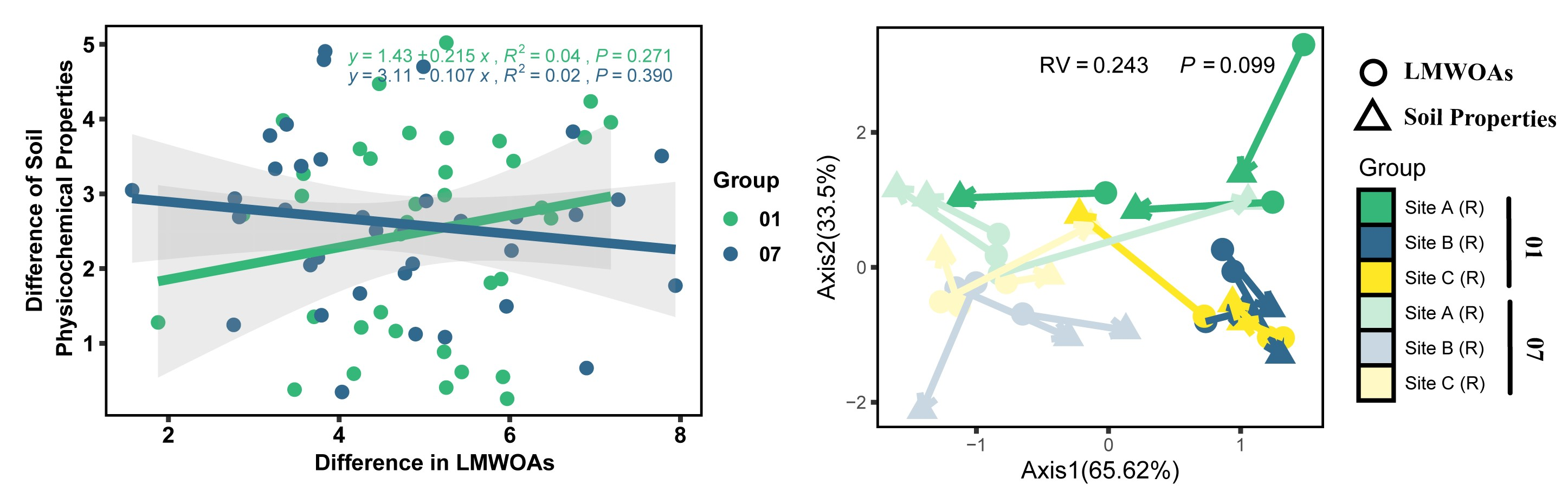


**Fig. S****3** **Linear regression and co-inertia analysis between** **LMWOAs and rhizosphere soil properties**. Site A: marsh center of *S. alterniflora*; Site B: marsh border of *S. alterniflora* neighboring *K. obovata*; Site C: marsh border of *S. alterniflora* neighboring *A. corniculatum*. (R): rhizosphere soil. 01: senescent period of *S. alterniflora*; 07: vigorous growth period of *S. alterniflora*.


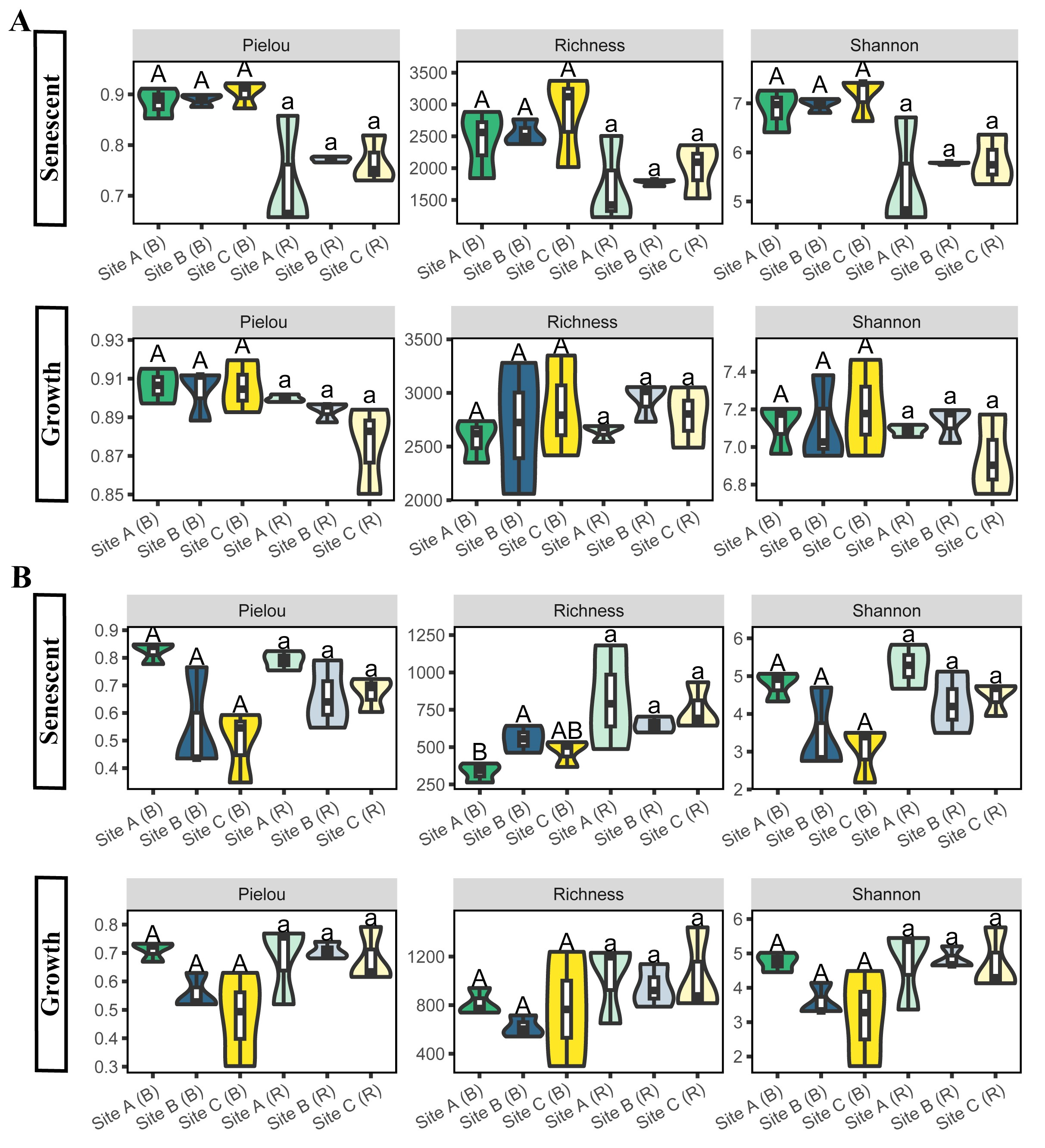


**Fig. S4 Alpha diversities of soil bacterial (A) and fungal (B) communities between different groups.** Different letters indicate the statistical difference between particular groups (*P* < 0.05). Site A: marsh center of *S. alterniflora*; Site B: marsh border of *S. alterniflora* neighboring *K. obovata*; Site C: marsh border of *S. alterniflora* neighboring *A. corniculatum*. (R): rhizosphere soil; (B): bulk soil.


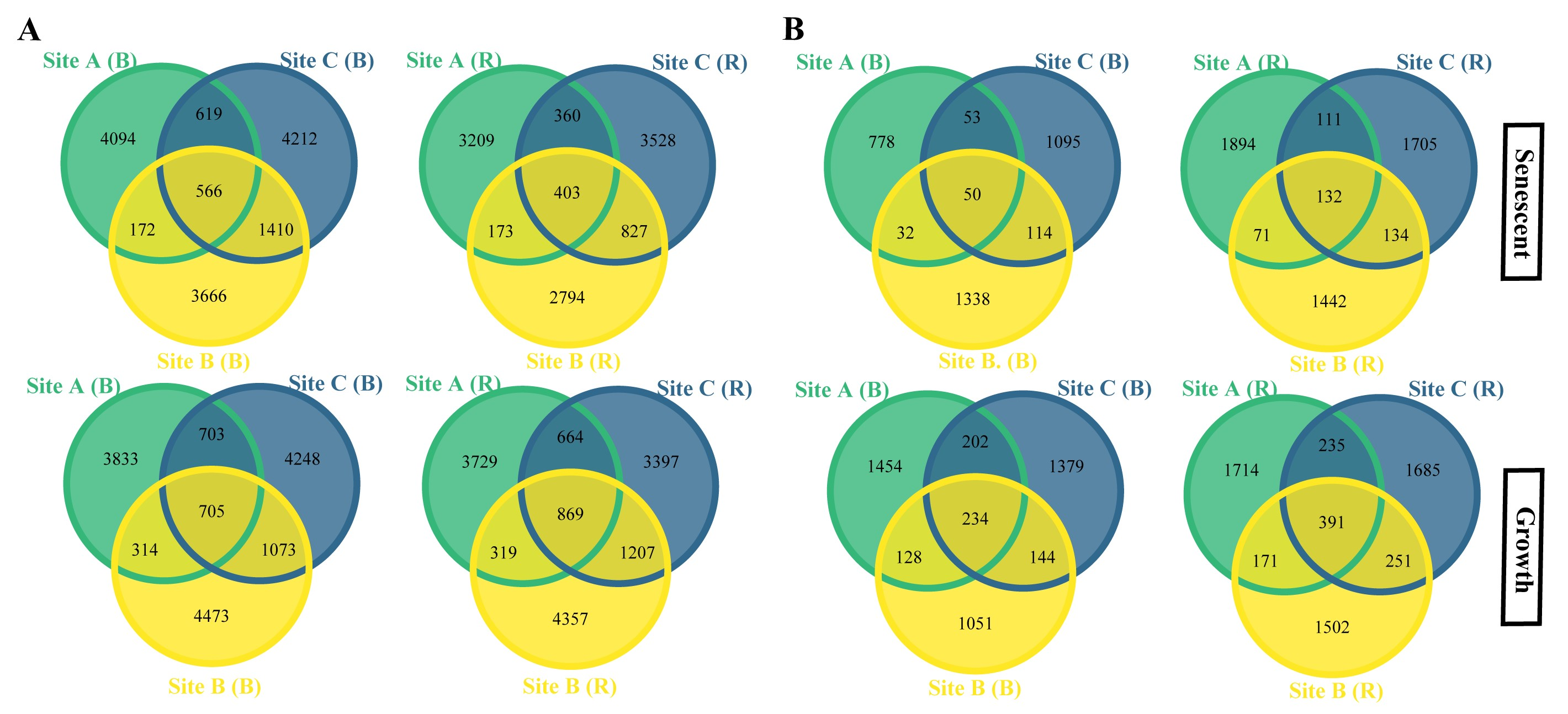


**Fig. S5 Venn diagrams showing overlapped bacterial (A) and fungal (B) ASVs in rhizosphere and bulk soils among different groups** **in senescent and growth periods of *S. alterniflora*.** Site A: marsh center of *S. alterniflora*; Site B: marsh border of *S. alterniflora* neighboring *K. obovata*; Site C: marsh border of *S. alterniflora* neighboring *A. corniculatum*. (R): rhizosphere soil; (B): bulk soil.


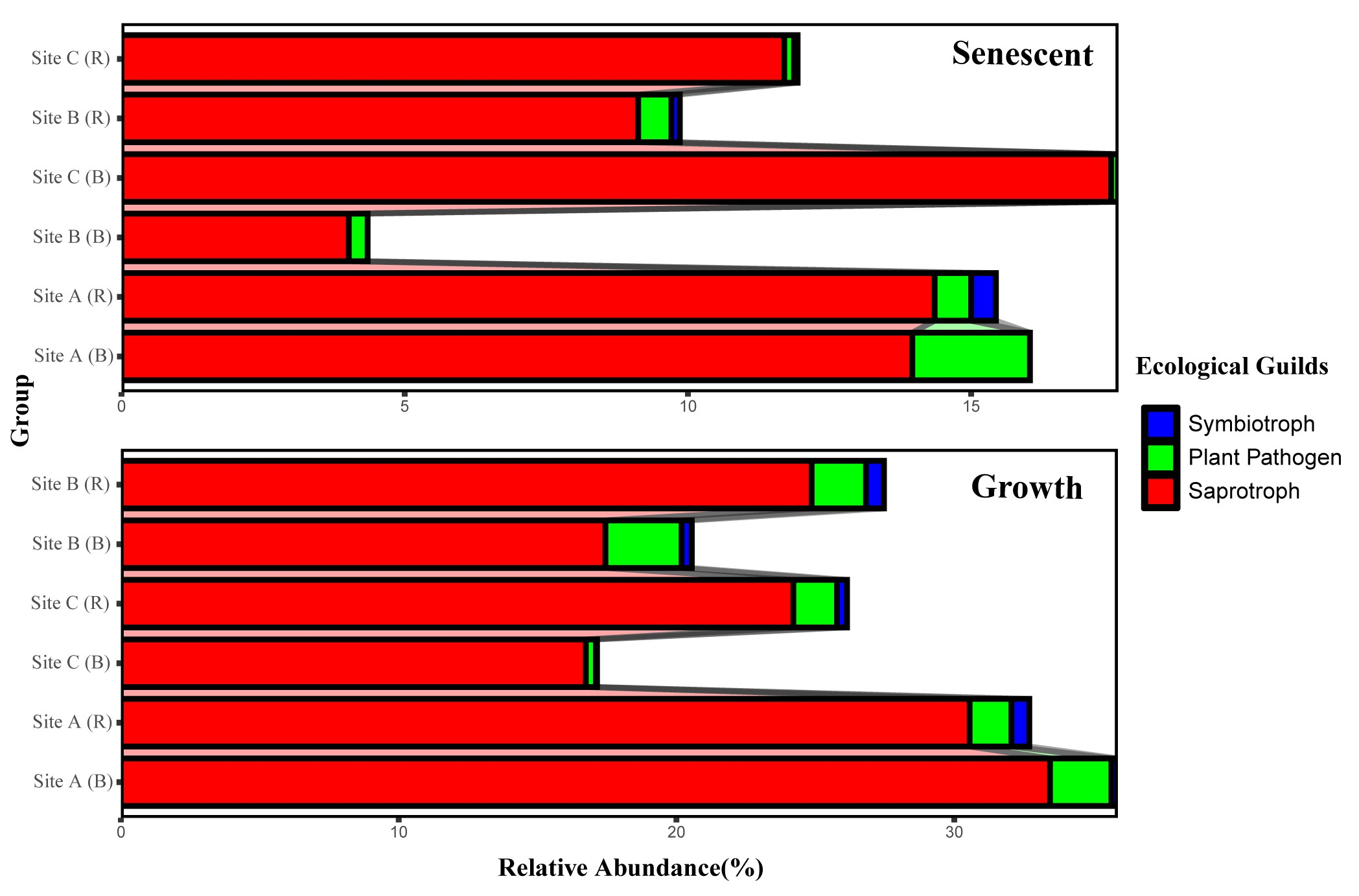


**Fig. S6 Functional guild composition of fungi in rhizosphere and bulk soils of *S. alterniflora* in different groups.** Site A: marsh center of *S. alterniflora*; Site B: marsh border of *S. alterniflora* neighboring *K. obovata*; Site C: marsh border of *S. alterniflora* neighboring *A. corniculatum*. (R): rhizosphere soil; (B): bulk soil.


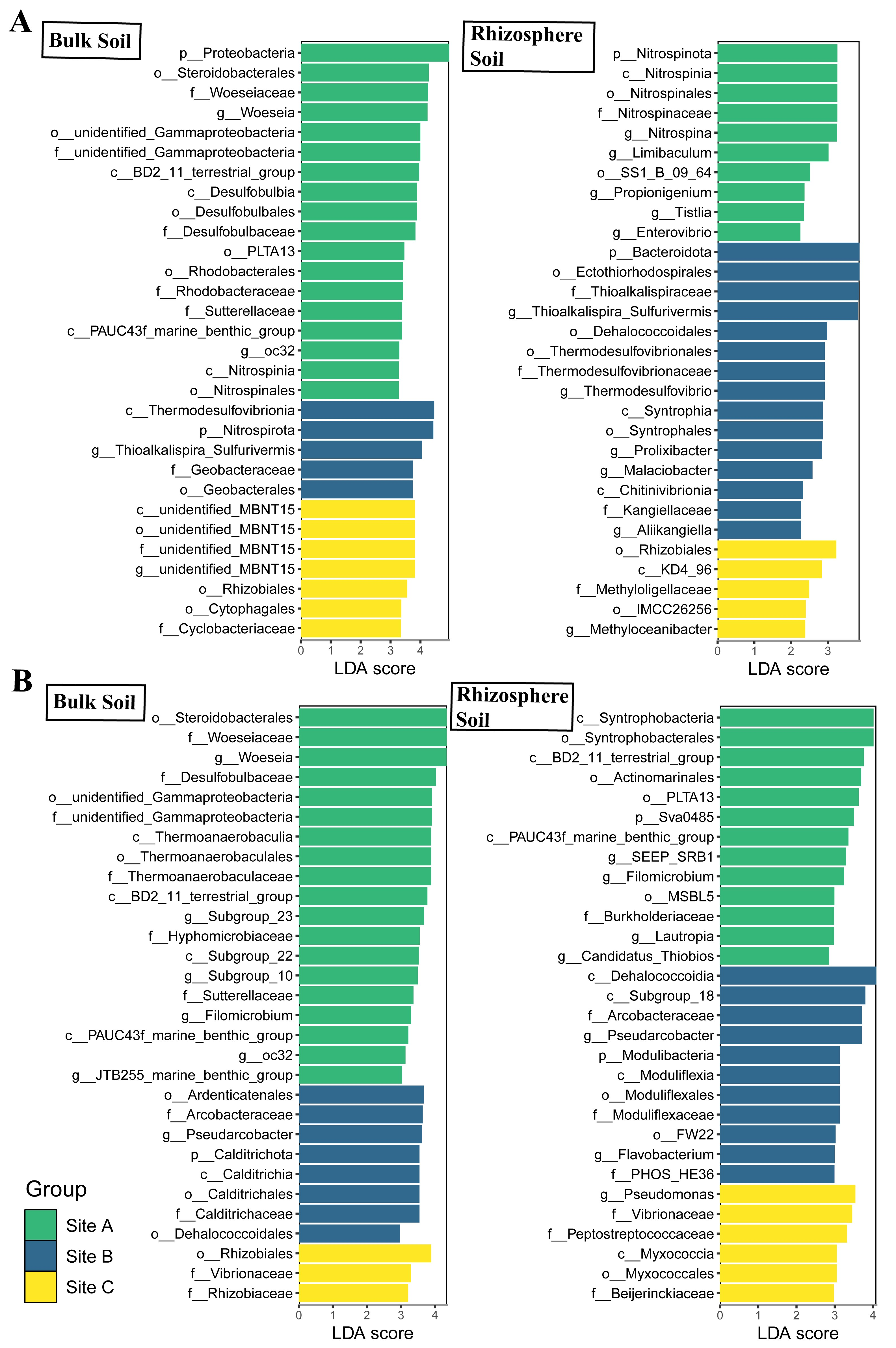


**Fig. S7** **Bacterial biomarkers** **in rhizosphere and bulk soils of *S. alterniflora* in different groups were identified via linear discriminant analysis effect size (LEfSe) analysis in senescent (A) and vigorous growth (B) periods.** Site A: marsh center of *S. alterniflora*; Site B: marsh border of *S. alterniflora* neighboring *K. obovata*; Site C: marsh border of *S. alterniflora* neighboring *A. corniculatum*.


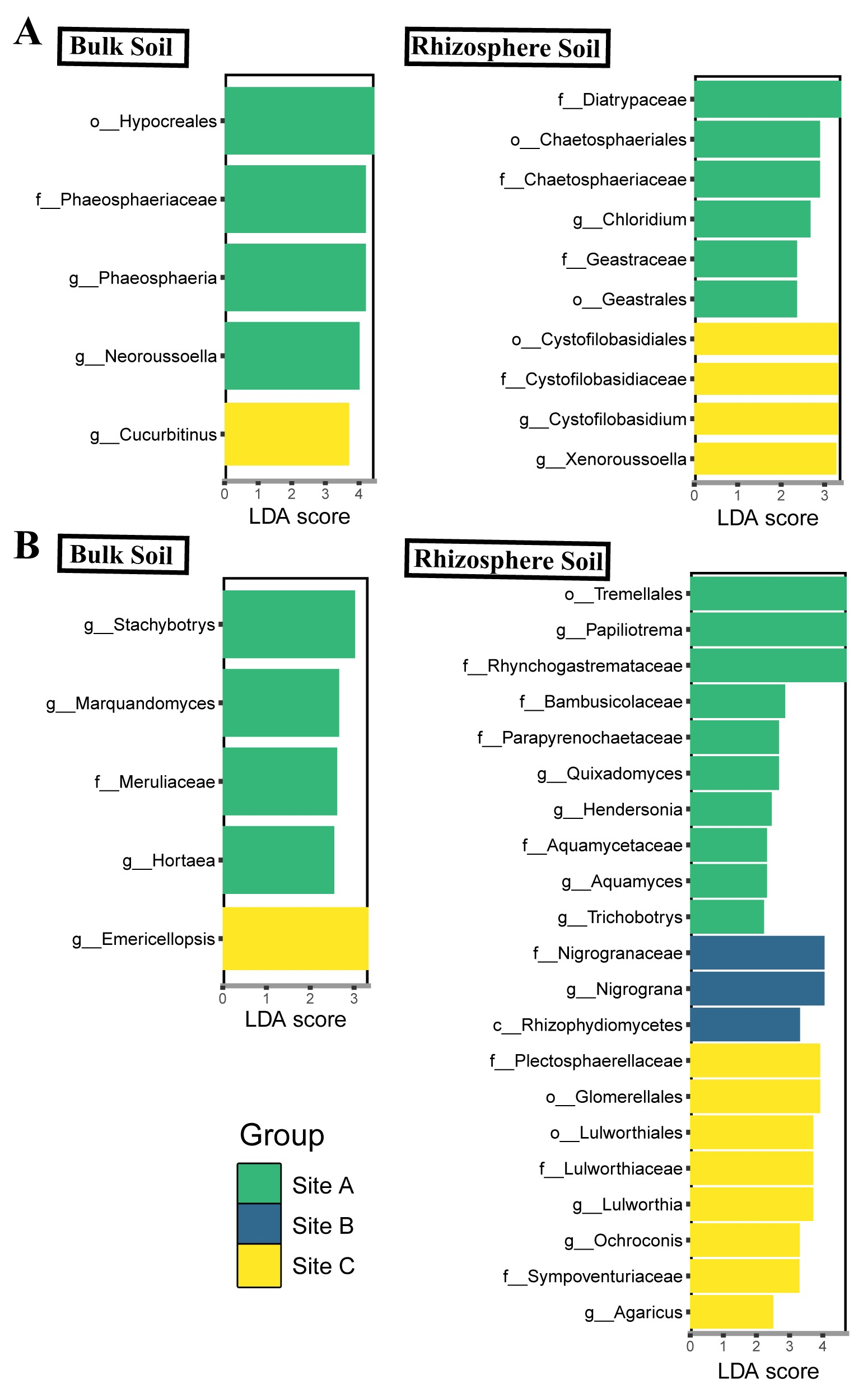


**Fig. S8 Fungal biomarkers in rhizosphere and bulk soil of *S. alterniflora* in different groups were identified via LEfSe analysis in senescent (A) and vigorous growth (B) periods.** Site A: marsh center of *S. alterniflora*; Site B: marsh border of *S. alterniflora* neighboring *K. obovata*; Site C: marsh border of *S. alterniflora* neighboring *A. corniculatum*.


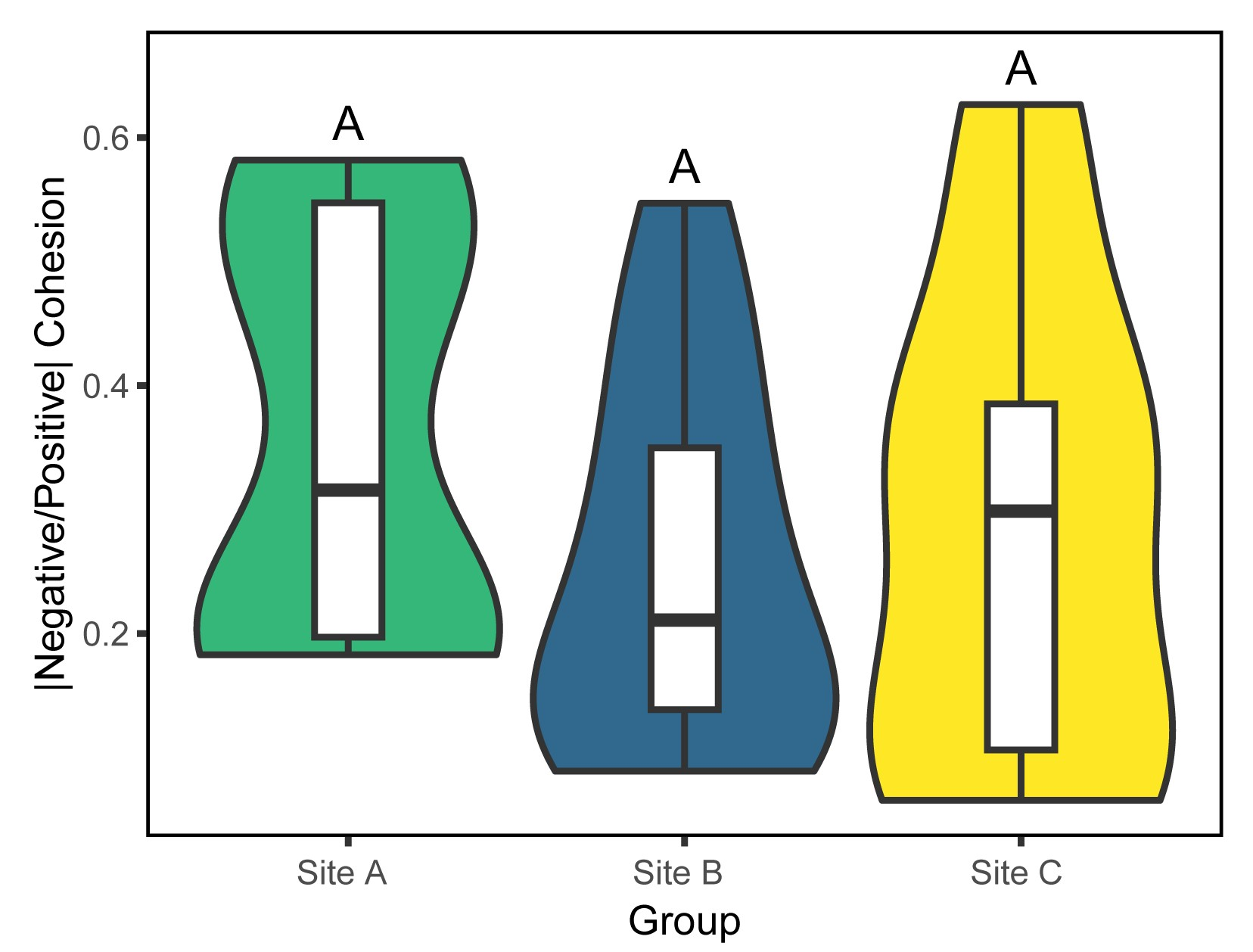


**Fig. S9 Absolute value of the ratio of negative: positive cohesion of different groups.** Site A: marsh center of *S. alterniflora*; Site B: marsh border of *S. alterniflora* neighboring *K. obovata*; Site C: marsh border of *S. alterniflora* neighboring *A. corniculatum*.


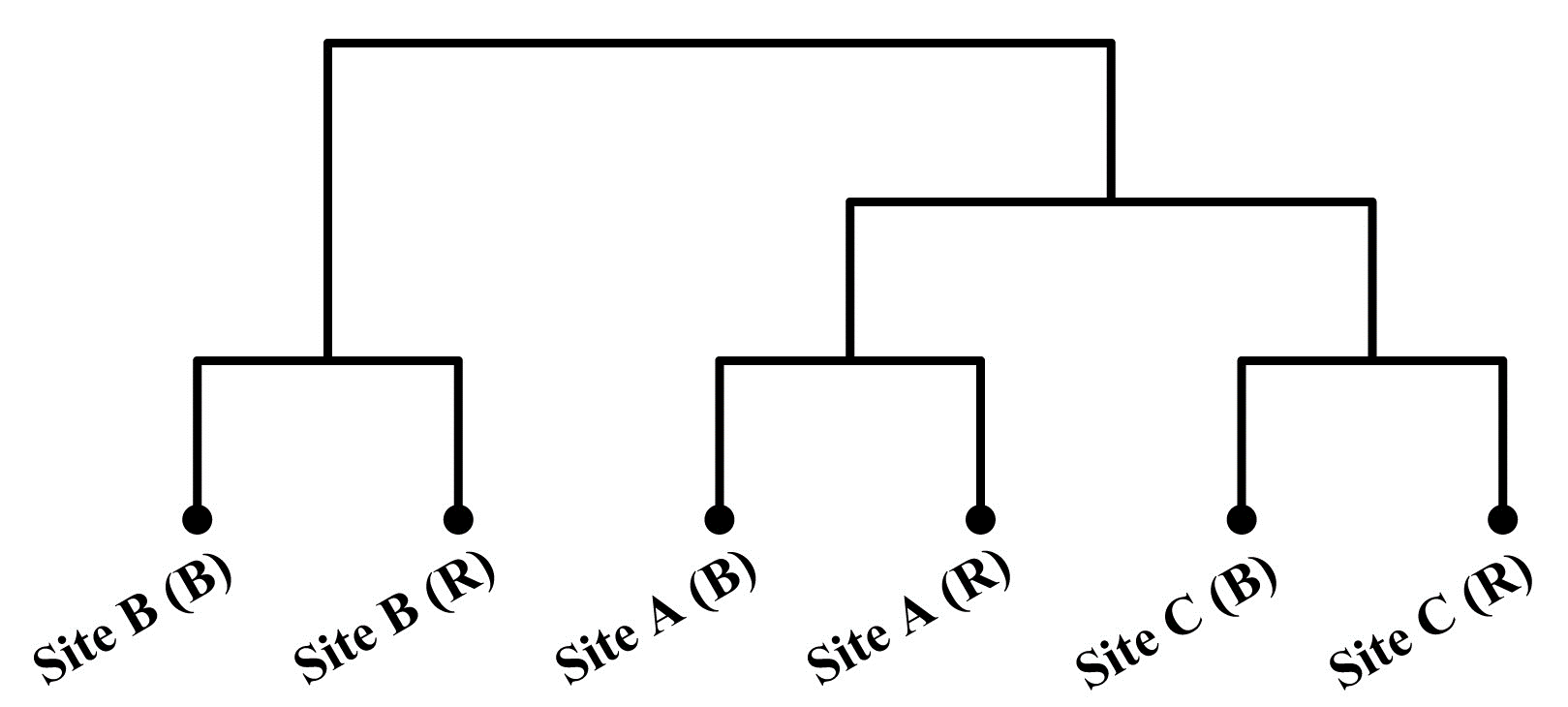


**Fig. S10 cluster analysis basing on total functional genes (KEGG level4).** Site A: marsh center of *S. alterniflora*; Site B: marsh border of *S. alterniflora* neighboring *K. obovata*; Site C: marsh border of *S. alterniflora* neighboring *A. corniculatum*. (R): rhizosphere soil; (B): bulk soil.


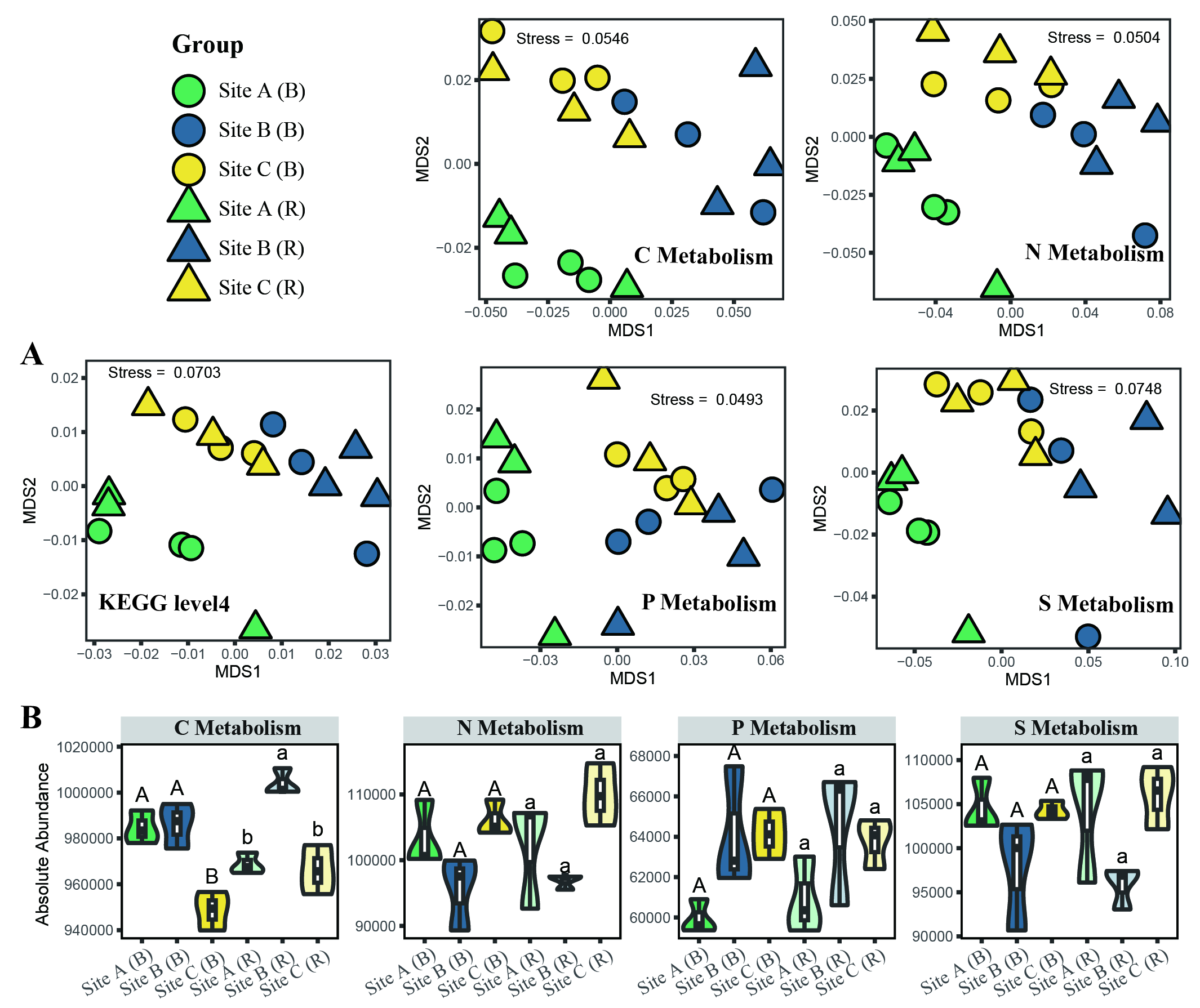


**Fig. S11 The variation of microbial functional genes induced by plant interspecific interactions.** Nonmetric multidimensional scaling (NMDS) of microbial functional genes (A). The normalized abundances (B) of functional genes involved in C, N, P, and S metabolisms, respectively. Different letters indicate the statistical difference between particular groups (*P* < 0.05). Site A: marsh center of *S. alterniflora*; Site B: marsh border of *S. alterniflora* neighboring *K. obovata*; Site C: marsh border of *S. alterniflora* neighboring *A. corniculatum*. (R): rhizosphere soil; (B): bulk soil.


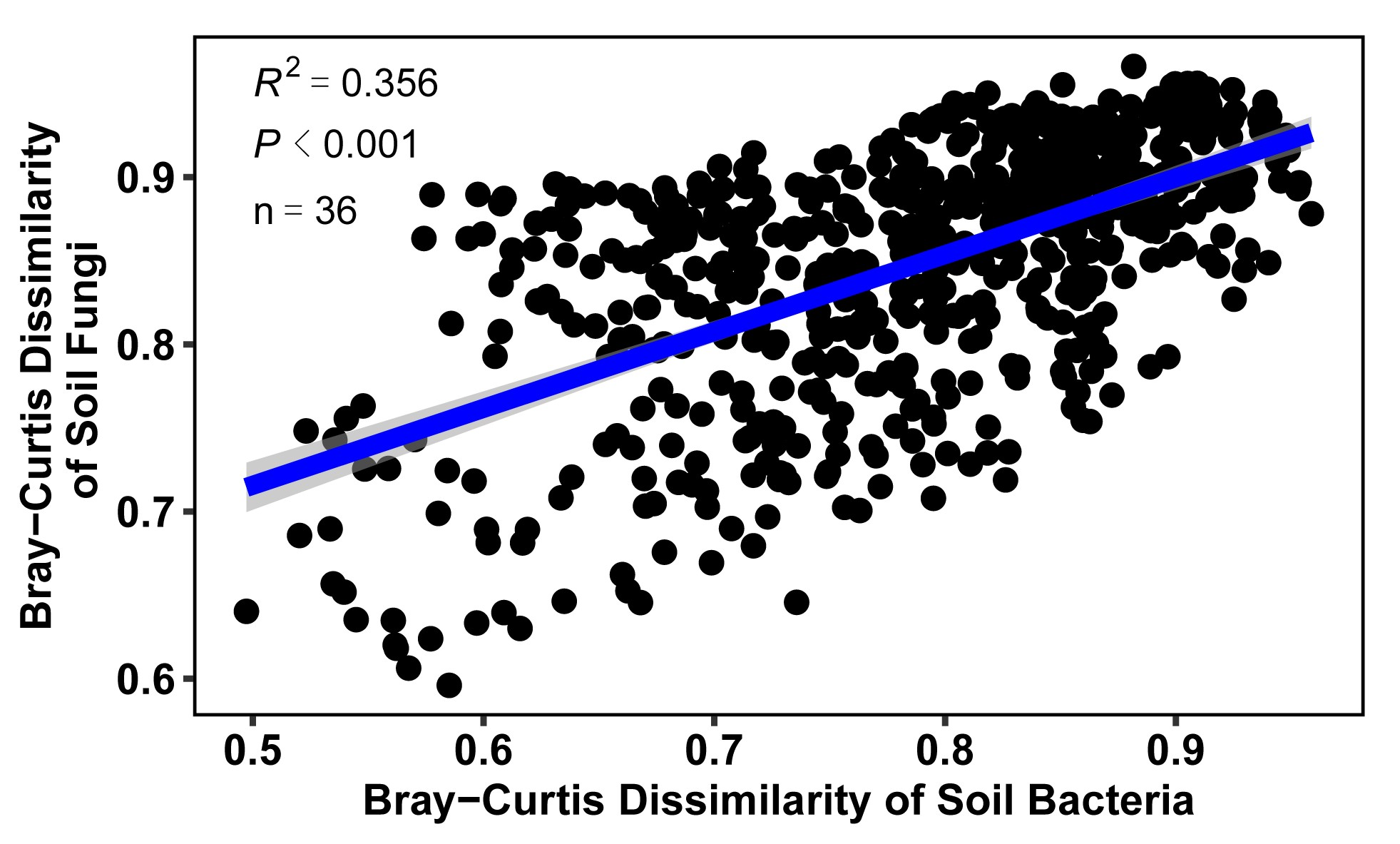


**Fig. S12 Linear relationship between soil bacterial and fungal communities.**
